# The floral ABCs of *Hydnora,* one of the most bizarre parasitic plants in the world, and its autotrophic relatives of the order Piperales

**DOI:** 10.1186/s13227-025-00252-8

**Published:** 2025-10-02

**Authors:** Natalia Pabón-Mora, Favio González, Claude W. dePamphilis, Jay F. Bolin, Christoph Neinhuis, Juan F. Alzate, Stefan Wanke

**Affiliations:** 1 Instituto de Biología, Universidad de Antioquia, 050010 Medellín, Colombia; 2 Universidad Nacional de Colombia, Sede Bogotá, Facultad de Ciencias, Instituto de Ciencias Naturales, 111321 Bogotá, Colombia; 3 Department of Biology and Institute of Molecular Evolutionary Genetics, The Pennsylvania State University, University Park, PA USA; 4 Department of Biology, Catawba College, Salisbury, NC USA; 5 Technische Universität Dresden, Fakultät Biologie, 01062 Dresden, Germany; 6 Centro Nacional de Secuenciación Genómica - CNSG, Sede de Investigación Universitaria – SIU, Facultad de Medicina, Universidad de Antioquia, 050010 Medellín, Colombia; 7 Goethe University Frankfurt, Institute of Ecology, Evolution & Diversity, 60438 Frankfurt, Germany; 8 Senckenberg Forschungsinstitut und Naturmuseum, Botanik und Molekulare Evolutionsforschung, 60325 Frankfurt, Germany; 9 Departamento de Botánica, Universidad Nacional Autónoma de México, 04510 Mexico City, Mexico

**Keywords:** Aristolochiaceae, Floral MADS-box genes, *Hydnora*, Holoparasitism, Perianth evolution

## Abstract

*Hydnora* (Hydnoraceae) comprises a few parasitic species exceptional among the autotrophic members of the perianth-bearing Piperales. Flowers in the genus are thick, fleshy, sapromyophilous, and develop into massive, polyspermous fruits. They are formed directly along underground rhizomes that parasitize species of Euphorbiaceae and Fabaceae. Due to its peculiar floral morphology and lack of leaves, *Hydnora* is often dubbed ‘the strangest plant in the world’. Here we generated the first transcriptomes of *Hydnora visseri* from the dissected rhizome, perianth, osmophore, stamen, carpel, and fruit. Our results suggest that *Hydnora* possesses one of the simplest developmental genetic toolkits for flowering and floral organ identity among angiosperms, further emphasizing its uniqueness. We detected that most of the photoperiodic flowering integrators are expressed. In contrast, regulators of the autonomous pathway and circadian clock were notably absent from the transcriptomes. Conversely, we identified an intact genetic toolkit linked to floral organ identity and fruit development in *Hydnora*. Through positional homology and gene expression data, we inferred that the perianth of *Hydnora* corresponds to the calyx, and that the osmophores are late sepal elaborations. Additionally, the expression patterns of genes responsible for stamen, carpel, and ovule identity align with the canonical ABCDE model. Finally, we recorded large-scale duplications in putative perianth identity genes prior to the diversification of all perianth-bearing Piperales. This study serves as an additional comparative point for assessing the evolutionary onset of holoparasitic plants, as *Hydnora* and its sister genus *Prosopanche* are likely the earliest branching representatives of this lifestyle across angiosperms.

**Supplementary Information:**

The online version contains supplementary material available at 10.1186/s13227-025-00252-8.

## Introduction

With over 300,000 species, flowering plants have successfully colonized almost all terrestrial and shallow aquatic environments on Earth. Although monocots and eudicots account for 23% and 70% of Angiosperm diversity, respectively, the remaining 7% exhibits the highest floral disparity [67, 71, 94, 95]. Flowering (i.e., the reproductive transition) and the acquisition of floral organ identity are two key developmental and evolutionary processes in angiosperms; thus, their understanding in early diverging lineages is crucial to assess the origin and diversification of flowers. MADS-box transcription factors are major hubs in these processes, along with other critical developmental transitions including gametogenesis, fertilization, embryogenesis, vegetative growth, and fruit patterning [5, 79]. However, MADS-box genes have been predominantly studied in model core-eudicot and monocot species, largely neglecting the plethora of flower diversity in early diverging angiosperms. Therefore, exploring the evolution, gene expression, and function of these pivotal genes in members of the ANA grade and in magnoliids is critical for a better understanding of how early flowers were structured and how they attained such remarkable diversity.

**Fig. 1:**
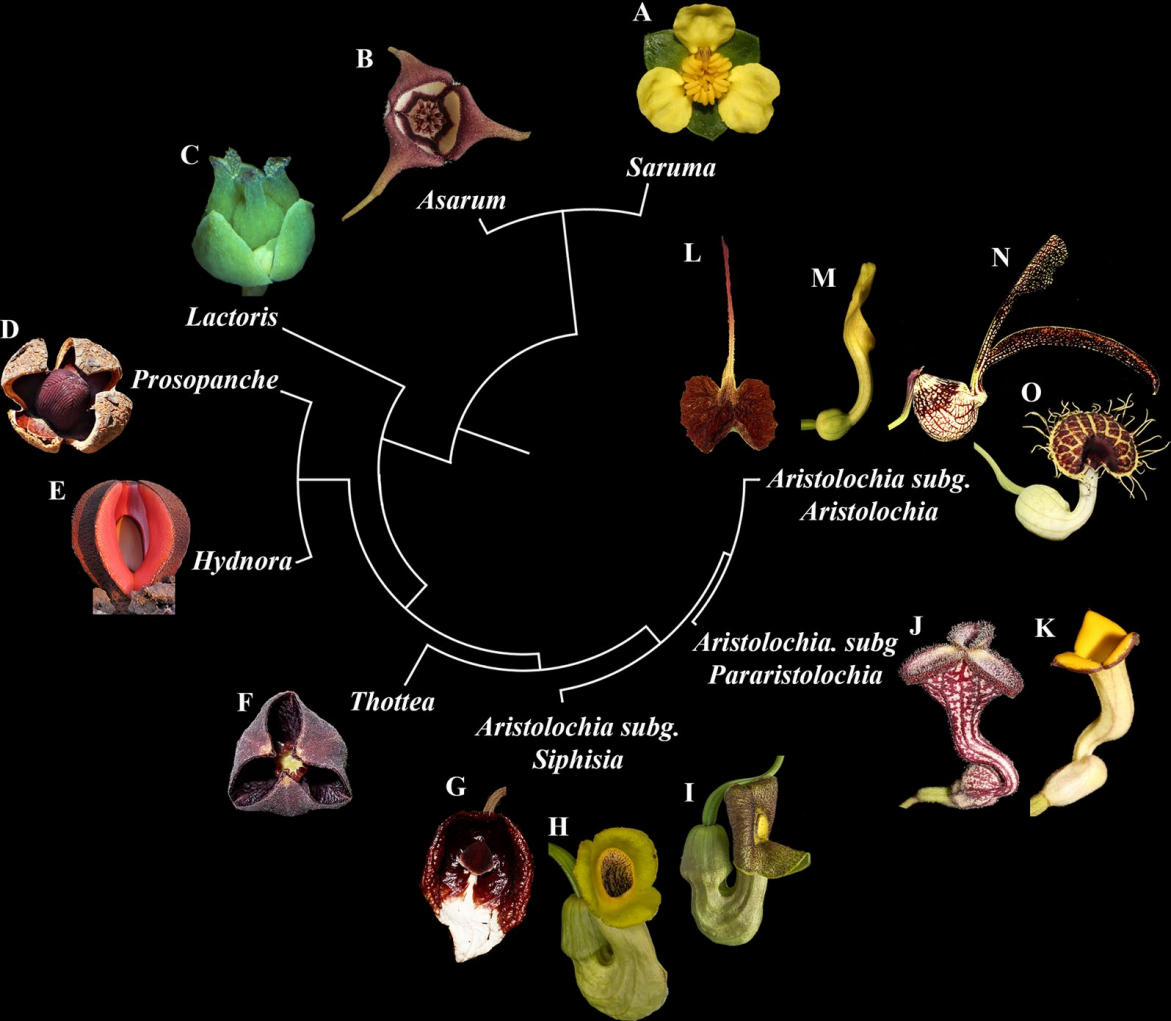
Outline of the phylogenetic relationships among genera of perianth-bearing Piperales (modified from [42], but see [37] for a different topology with Hydnoraceae sister to all other Piperales), with representative species, as follows: **A** *Saruma henryi*, **B** *Asarum canadense*; **C** *Lactoris fernandeziana* Phil.; **D** *Prosopanche americana* (R. Br.) Baill.; **E** *Hydnora visseri*; **F** *Thottea siliquosa*; **G**-**I** *Aristolochia* subg*. Siphisia*: (G) *A. arborea*; (H) *A. macrophylla*; (I) *A. manshuriensis*; **J**, **K** *Aristolochia* subg. *Pararistolochia* (J) *A. deltantha*; (K) *A. praevenosa*. **L**-**O** *Aristolochia* subg. *Aristolochia*: L *A. lindneri*; **M** *A. clematitis*; **N** *A. ringens*; **O** *A. fimbriata*. (Photo credits: A-C, F-O, F. González; D, G. Roget at iNaturalist CC BY-NC; E, S. Wanke)

Here we focus on Piperales, the most species-rich magnoliid order comprising ca. 4200 species in 16 genera that exhibit a remarkable diversity of growth forms and a wide assortment of floral ground plans (Fig. 1; Suppl. Table S1; [42]). Piperales consist of two clades with highly divergent floral traits, namely, the perianth-less Piperaceae and Saururaceae, and the perianth-bearing Aristolochiaceae, Asaraceae, Lactoridaceae, and Hydnoraceae. The most consistent floral trait of the latter clade is the trimerous perianth. Specifically, the clade comprises Asaroideae, with the genera *Saruma*, one species native to China, and *Asarum*, with ca. 130 spp. in North America and Asia plus one species in Europe; [77], Lactoridaceae, with the monospecific genus *Lactoris*, from Juan Fernandez Islands, Chile [27], Hydnoraceae with the genera *Hydnora* (seven species from Africa, Madagascar and the Arabian Peninsula) and *Prosopanche* (seven species from Central- and South America) [35, 58] and Aristolochioideae with two genera, the south-eastern Asian *Thottea* (ca. 46 spp. in tropical Asia and the Indo-Pacific region) [64], and the widespread genus *Aristolochia* (ca. 600 spp.) [91].

**Fig. 2:**
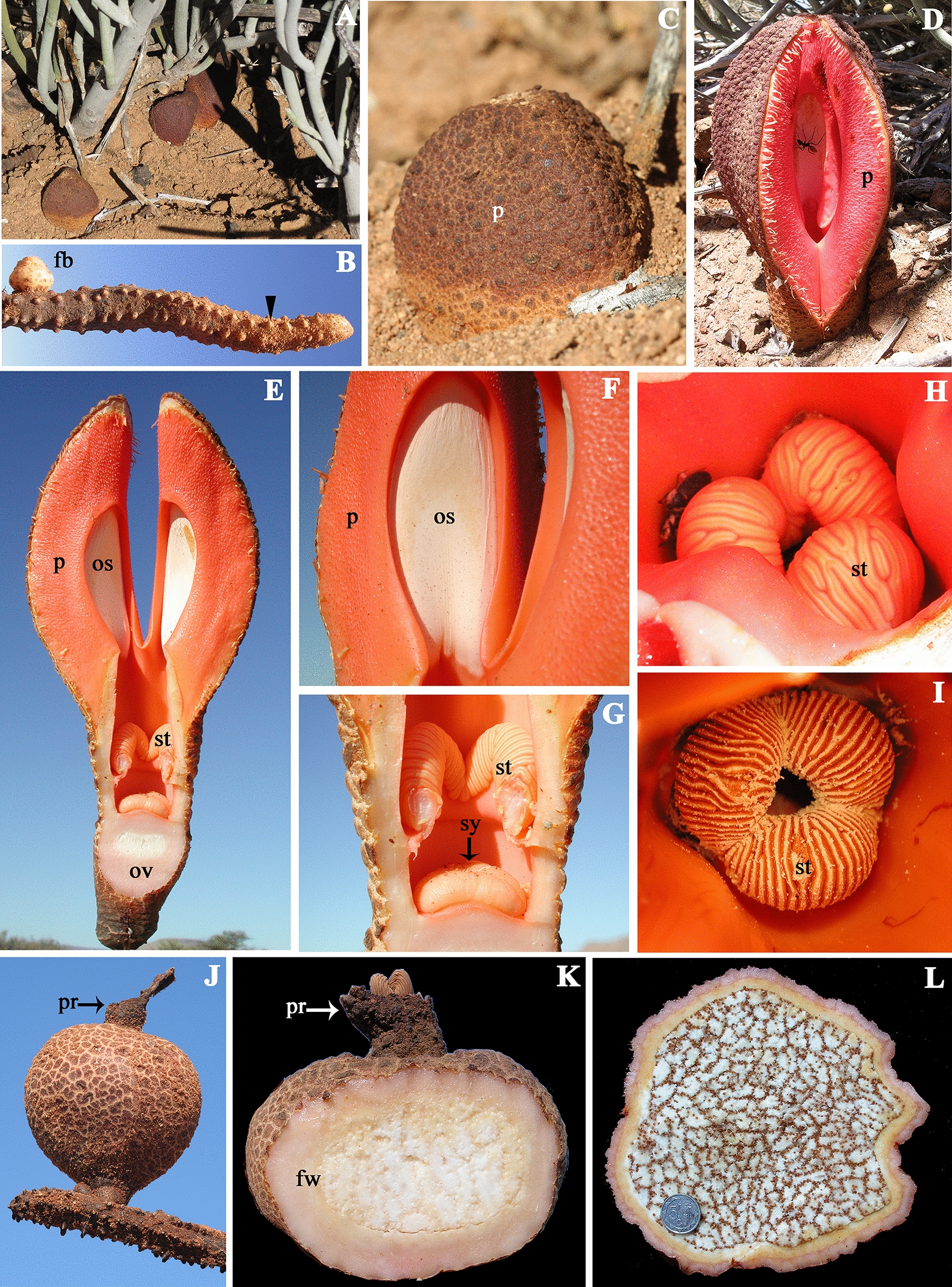
*Hydnora visseri*. **A**. Emerging floral buds at different developmental stages growing on its host *Euphorbia gregaria* at Gondwana Cañon Preserve, Namibia*.* **B**. Rhizome with floral bud and warty protuberances (arrowhead). **C, D**. Flowers at preanthesis (**C**) and anthesis (**D**) exhibiting only the uppermost portion of the perianth. **E**. Longitudinal section of flower at anthesis showing the inferior ovary, the stamens in the tubular region, and two perianth lobes carrying the osmophores. **F**. Detail of the recessed osmophores. **G**. Detail of the stamens above the massive stigma. **H**–**I**. Immature (**H**) and mature (**I**) stamens. **J**. Fruit. **K**. Longitudinal section of young fruit. **L**. Transverse section of mature fruit. Abbreviations: fb, floral bud; fw, fruit wall; os, osmophores; ov, ovary; p, perianth; pr, perianth remnants; st, stamens; sy, stigma

The trimerous perianth in *Saruma* is either biseriate and sharply differentiated into three green sepals and three bright yellow petals or uniseriate and sepal-derived in *Aristolochia, Asarum, Hydnora*, *Lactoris, Prosopanche*, and *Thottea* [27, 29, 35, 36] (Fig. 1). Fusion of perianth organs occurs in Hydnoraceae and Aristolochiaceae (Fig. 2; [7–9, 33, 35, 36, 54, 58, 76, 86, 87]) (Fig. 2). *Aristolochia* flowers exhibit a typical bilateral perianth that differentiates into an inflated basal utricle, a connecting narrow tube, and a distal, expanded limb [29, 30, 67, 80–82]. Conversely, the merosity, morphology, and synorganization of the androecium and the gynoecium, as well as the fruit type, are highly variable across perianth-bearing Piperales (Suppl. Table S1).

The recent inclusion of Hydnoraceae in the perianth-bearing Piperales adds holoparasitism as an additional, yet exceptional, lifestyle to a plant group where autotrophic herbs, shrubs, vines, and lianas predominate [37, 59, 62]. Holoparasitic plants exhibit some of the most striking vegetative reductions, yet maintain the typical reproductive transition, ovule, and fruit development, with respect to their most closely related autotrophic plants. Within perianth-bearing Piperales, extremely elaborated floral morphologies have evolved in both lifestyles, that is, the autotrophic *Aristolochia* and the holoparasitic Hydnoraceae. Flowers in Hydnoraceae emerge attached to an underground leafless runner (Fig. 2a, b), which raises the interesting question of how flowering and floral development occur without typical vegetative organs. Their flowers develop below or above ground, seasonally bearing a trimerous and massive perianth that is brownish on the outside and orange-reddish on the inside (Fig. 2c, d). The inner surface has fleshy pads representing osmophores, which emit fetid odors to attract pollinators ([86]; Fig. 2e, f). The stamens and carpels occupy different levels in the narrow tubular region of the flower (Fig. 2e, g). Stamens are polysporangiate (Fig. 2h, i). Fruits are derived from inferior ovaries and break open through irregular slits (Fig. 2j). The placenta is massive, and seeds are minute (Fig. 2k, l). Their overall appearance has granted *Hydnora* the title of “the strangest plants in the world” (Fig. 2; [58]). Such striking and highly elaborate morphology merits an in-depth investigation of the genetic bases of flowering transition, as well as floral organ identity.

Here, we aimed to examine in detail the evolution of critical floral MADS-box genes across the perianth-bearing Piperales, considering that: (1) they encompass a remarkable diversity in terms of life forms (including holoparasitism) and floral ground plans; (2) members of Hydnoraceae likely represent the first evolutionary event of holoparasitism in angiosperms [59], and (3) Hydnoraceae flowers exhibit remarkably atypical morphologies whose homology assessment has been extensively debated. We target type II MIKCc MADS-box transcription factors, with special emphasis on *Hydnora* as, together with *Prosopanche*, they are the only holoparasitic lineages of Piperales, magnoliids and the ANA-grade taxa. Thus, our goal is to identify and evaluate the expression of flowering and floral organ identity MIKCc MADS-box genes of *Hydnora* and to compare them with those of its autotrophic relatives. In addition, considering that holoparasitic plants have extremely modified vegetative growth lacking leaves, which are the organs that sense and integrate flowering signals, we want to assess if the flowering genetic regulatory network in *Hydnora* has converged in the predominant expression of few dominant flowering pathways, like the photoperiod response, identified in other parasitic plants studied to date. Finally, we discuss conserved floral regulators consistently found in the disparate flowers of *Aristolochia, Asarum, Hydnora*, *Saruma* and *Thottea*.

## Materials and methods

***Flower terminology.*** We have followed Vaccaneo [86], Harms [33], Visser [87], Musselman and Visser [58], Meijer [7–9, 54], Seymour et al. [76] and Hatt et al. [35, 36] for *Hydnora*, Harms [33], Ruiz Leal (1950), Burkart [12], Cocucci [16–19], Rossi [73], Meijer [54], Cocucci and Cocucci [20], Vogt [88], Sato [75], and Martel et al. [53] for *Prosopanche*, Skottsberg [78], Carlquist [14], Bernardello et al. [6], and González and Rudall [27, 28] for *Lactoris*; and Griffith [31], González and Stevenson [29, 30], Pabón-Mora et al. [65–67] for *Aristolochia, Asarum, Saruma*, and *Thottea*. We describe the ovary in *Asarum* and *Saruma* as ‘half inferior’ in reference to the halfway placement of perianth organs and stamens with respect to the carpels in a fully formed flower.

**Field collection and assemblage of a reference transcriptome of Hydnora visseri Bolin, E. Maass & Musselman.** Plant tissues of *H. visseri* growing on its host plants *Euphorbia gregaria* Marloth. were collected in the Gondwana Cañon Preserve, Namibia under the MET Permit No. 1350/2009. Fresh individual tips of the growing rhizome, and emerging (1–2 cm long) and growing (5–6 cm long) floral buds were collected in the morning. The latter floral buds were dissected to separate perianth, osmophore tissue, androecium, and gynoecium. All samples and dissections were carefully made avoiding host tissues. Additionally, developing fruits and seeds were collected. Material was rinsed with nanopure water and peeled (if needed) to avoid contamination. Samples were placed in individual falcon tubes and flash-frozen in liquid nitrogen, shipped in a MVE cryo-shipper, and stored at − 80 °C. Individual samples and tissues were processed very quickly upon arrival to the lab. RNA was isolated following Cheng’s et al. (1993) CTAB protocol. The Ambion Plant Isolation Aid solution (AM9690) was used to remove polysaccharides and polyphenolics, as they decrease the RNA yield. Total RNA quantity and quality were determined by micro capillary electrophoresis using the Agilent BioAnalyzer (RNA 6000 Nano kit). To avoid DNA contamination, the RNA was treated with DNase even if there was no DNA visible in the BioAnalyzer run. After DNase treatment, RNA quality and quantity were checked again on the BioAnalyzer (RNA 6000 Nano kit). Libraries were built using the “Preparing Samples for Sequencing of mRNA” kit (Illumina RS-100-0801) using poly-T oligo-attached magnetic beads and fragmented using chemical fragmentation. For the subsequent generation of the cluster on the flow cell surface, we used the “Single-Read Cluster Generation Kit” (Illumina GD-203-2001). The immobilized sstDNA fragments were amplified via solid-phase bridge amplification, which results in dense clusters of target clones.

All samples were sequenced independently. Transcriptome assemblies follow [66]. Stats for the transcriptome assemblies of *Hydnora visseri* dissected organs and the global transcriptome built from all samples can be found in Supplementary Table S2 and are deposited at NCBI under bio-accessions PRJNA1111920, SAMN41406291: Hyd-Fruit, SAMN41406292: Hyd-Gynoecium, SAMN41406293: Hyd-Osmophore, SAMN41406294: Hyd-Rhizome, SAMN41406295: Hyd-Stamens, SAMN41406296: Hyd-Tepals. The metrics for the global transcriptome are within the range of expected values for all other perianth-bearing Piperales indicating good coverage and depth (see below). We used transdecoder to identify the most likely contigs of *H. visseri* (https://github.com/TransDecoder/TransDecoder), and eggnog-mapper (http://eggnog-mapper.embl.de/) for annotation. A total of 59,921 peptides were annotated (Supplementary Table S3). In addition, all transcription factors were annotated using the plant transcription factor database (https://planttfdb.gao-lab.org/; Supplementary Table S4).

**Reference transcriptomes for non-parasitic perianth-bearing Piperales.** Reference transcriptomes used here correspond to those de novo assembled by [66], including two species of *Asarum* (*A. canadense* L. and *A. europaeum* L.), one of *Saruma* (*S. henryi* Oliv.) and one of *Thottea* (*T. siliquosa* (Lam.) Ding Hou). Transcriptomes for seven species from all three subgenera of *Aristolochia* are available from the same study. These are: *A. clematitis* L. and *A. lindneri* A. Berger, from subgenus *Aristolochia*,*A. deltantha* F. Muell. and *A. praevenosa* F. Muell., from subgenus *Pararistolochia*; and *A. arborea* Linden., *A. macrophylla* Lam. and *A. manshuriensis* Kom., from subgenus *Siphisia*. Two additional previously available [67, 81] transcriptomes from *A. fimbriata* and *A. ringens* (both members of subgenus *Aristolochia*, were also included in the present study. The material was gathered from the living collections of the Arnold Arboretum at Harvard University (Roslindale, MA, USA, the Botanical Garden of the University of Technology Dresden (Dresden, Germany, and the Botanical Garden of Medellín (Colombia; Supplementary Table S5). These reference transcriptomes were obtained from a mix of all aboveground organs, including young shoot apical meristems and flanking leaves, inflorescences, flowers at different stages of development, and young fruits (if available). All organs and tissues were mixed for RNA extraction as the goal was to have a reference mixed transcriptome to isolate as many expressed MADS-box genes as possible. The reference transcriptomes are only used to isolate sequences and not for quantitative gene expression analyses.

Tissues from non-parasitic perianth-bearing Piperales were flash-frozen in liquid nitrogen and stored at − 80 °C until further processing. Total RNA from each species was extracted using TRIzol reagent (Invitrogen) or TRIsure (Meridian Life Science Inc., Memphis, TN, USA), resuspended in 1 ml of 100% ethanol and sent to the sequencing facility. The RNA-seq experiment was conducted using the Truseq mRNA library construction kit (Illumina) and sequenced on a HiSeq2000 instrument reading 100 bases, paired-end reads. Read cleaning was performed with a quality threshold of Q30 and a minimum read length of 70 bases using the TRIMMOMATIC flag. Contig assembly was computed using the TRINITY package following default settings. Standard metrics for each transcriptome were calculated (Supplementary Table S5).

**Targeted search and phylogenetic analyses of MADS-box homologues in Piperales.** In order to isolate putative MIKC^c^ MADS-box gene homologs in our generated transcriptomes, previously reported sequences from *Amborella trichopoda, Arabidopsis thaliana*, and *Nymphaea thermarum* were used as queries. These included the canonical members of *A. thaliana AGAMOUS* (*AG*), *AGAMOUS-LIKE 6 (AGL6)*, *AGL14*, *AGL15*, *AGL16*, *AGL17*, *AGL18*, *AGL19*, *AGL21*, *AGL24*, *AGL42*, *AGL71*, *AGL72*, *AGL79*, *ARABIDOPSIS B-SISTER* (*ABS*), *APETALA1* (*AP1*), *APETALA3* (*AP3*), *CAULIFLOWER* (*CAL*), *FLOWERING LOCUS C* (*FLC*), *FRUITFULL* (*FUL*), *GORDITA* (*GOA*), *PISTILLATA* (*PI*), *SEEDSTICK* (*STK*), *SEPALLATA1* (*SEP1*), *SEP2*, *SEP3*, *SEP4*, *SHATTERPROOF1* (*SHP1*), *SHP2*, *SHORT VEGETATIVE PHASE* (*SVP*), *SUPRESSORSUPPRESSOR OF CONSTANS* (*SOC1*), and *XAANTAL1* (*XAL1*). In addition, *TDR8*, a MADS-box gene lacking in Brassicaceae, yet well known in Solanaceae, was also included.

**Fig. 3:**
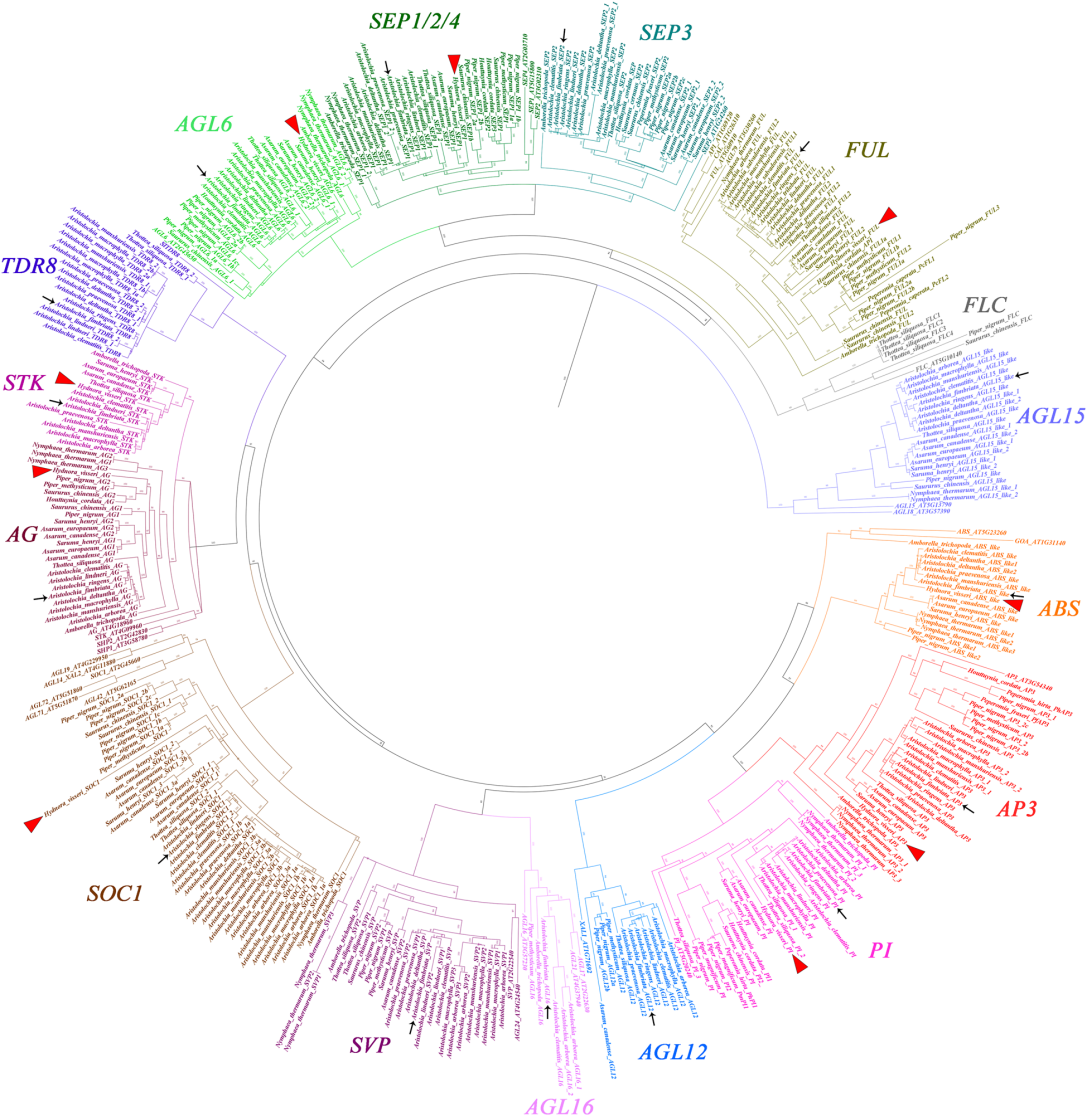
Phylogenetic reconstruction of the MIKCc Type II MADS-box genes in Piperales. Sampling includes all species studied in this research, plus genes from *Nymphaea* and the canonical MADS-box genes of *Arabidopsis thaliana*. Each gene lineage is differentially colored and labeled. Red arrowheads point to MADS-box homologs detected in the *Hydnora visseri* transcriptome. Arrows point to genes identified in *Aristolochia firmbriata*, the only member of the perianth-bearing Piperales with an annotated available genome to date

Searches were performed using TBLASTX tools [2] on all assembled contigs (Supplementary tables S2, S5). In addition, searches were made in the available genomes of *Aristolochia fimbriata* [71], *Piper nigrum* L. [39], and *Saururus chinensis* hort. ex Loudon [92], as well as for other Piperales species available in Genbank including *Houttuynia cordata* Thunb., *Peperomia caperata* Yunck., and *Piper methysticum* G.Forst. BIOEDIT (http://www.mbio.ncsu.edu/BioEdit/bioedit.html) was used to compile all isolated sequences and to manually identify open reading frames (ORF) and perform CDS prediction. Different versions of the same gene were retained as such when (1) they corresponded to different contigs or (2) they corresponded to the same contig but had significant variation. For the latter, significant changes included (1) premature stop codons, (2) more than 5% differences in their CDS nucleotide sequences, and (3) alternative splice forms. Large-scale (or ancient) gene duplications (affecting more than one species) were labeled as such if orthologs of two species or more fell into distinct clades. However, the assessment of species-specific copies versus allelic variation or any form of mRNA variation during and after transcription will need confirmation from genome sequencing in the future. Retention of different versions of the genes was preferred over reporting a single consensus sequence, as in other plant species MADS box alternative splicing forms have different expression patterns. A total of 350 nucleotide sequences from all previously mentioned Piperales species, as well as *Amborella trichopoda*, *Nymphaea thermarum*, and *Arabidopsis thaliana* were aligned using the online version of MAFFT (http://mafft.cbrc.jp/alignment/software/) [44] with a gap open penalty of 4.0, offset value of 1.0, and all other default settings. The alignment was then manually refined in BIOEDIT to ensure that the MIKC regions were comparable across sequences and that the reported motifs in each gene’s C-terminal domain were properly aligned. A second alignment was performed using protein data in MAFFT, which was then reverse translated to the corresponding nucleotides using the online version of PAL2NAL (https://www.bork.embl.de/pal2nal/) (Suyama et al., 2006). Phylogenetic analyses were performed using maximum likelihood (ML) with IQ-TREE [61]. The nucleotide substitution model that best fit our data was selected with MODELFINDER integrated in IQ-TREE [43]. Supports were estimated using the UltraFast Bootstrap (UFBS) method using 1000 pseudoreplicates [38]. The *Arabidopsis thaliana AtAGL66* and *AtAGL104* were used as outgroups. The tree was observed and edited using FIGTREE v.1.4.3 [72]. A complete circular tree is shown in Fig. 3. The newly isolated sequences from our transcriptomes were deposited under the NCBI submissions PX108523–PX108533, PX108544–PX108582, PX108584–PX108606, PX122869–PX122976. Duplications were mapped using stars in Photoshop. The expanded view of all subclades in the tree with mapped duplications is shown in Suppl Figs S1–S5.

**MADS-box expression analyses.** To estimate the relative abundance of the assembled contigs, cleaned reads were mapped against the de novo assembled dataset implementing the algorithm Kallisto v.0.46.0 with default settings (https://pachterlab.github.io/kallisto/). Kallisto quantifies transcript expression normalizing the relative abundance of each contig/transcript using the transcript per million (TPMs) metrics [11]. *ACTIN* expression was also included in the analysis, especially as there is a single biological sample of each floral organ and no biological replicates were collected. Heatmapper (http://www.heatmapper.ca/expression/, [4]) served to construct heat maps; however, as data come from a single sample, data are not strictly quantitative and should be interpreted with caution, in a more qualitative (presence/absence) manner. Asterisks point to samples where specific transcripts were not found (TPMs:0).

**Annotation of flowering regulation genes and integrators-.** We used a compiled list of flowering integrators known from *Arabidopsis thaliana* [49] to blast all genes involved in photoperiod, circadian clock, autonomous pathway, vernalization, and age that confirm the flowering genetic cascade in three selected species: *Aristolochia macrophylla, A. fimbriata*, and *Hydnora visseri*. *Aristolochia macrophylla* was used as a representative temperate species requiring vernalization to set flowers, whereas *A. fimbriata* was used as a subtropical taxon that does not require vernalization for flowering, and *H. visseri* represented a parasitic taxon from Namibian deserts. Presence and absence of a specific homolog were assessed by the coverage, percentage ID, blast best hit, and the e-value. If percentage ID was lower than 30% and/or coverage lower than 30%, the gene is labeled as “not found” in the targeted transcriptome. Only percentages over 70% and/or coverages over 45% were confidently marked as present (Supplementary tables S6, S7 and S8). We compared MADS box genes (Supplementary table S9) and all flowering integrators (Supplementary table S10) with all other published genomes and transcriptomes of holoparasitic plants [13, 25], Chen et al. (2023). Formal phylogenetic analyses to corroborate phylogenetic affinities in early flowering genes were made for *TOC1*, *PHYA*, *PHYB*, *PHYC*, and *CO* (Supplementary Figures S6–S8).

## Results

### Comparing MADS-box genes of the holoparasitic *Hydnora visseri* and autotrophic Piperales

The phylogenetic analyses of all MIKC^c^ MADS-box genes comprised genes from *Amborella trichopoda* Baill., *Nymphaea thermarum* Eb. Fisch., and representative members of perianth-bearing Piperales, including the holoparasitic *Hydnora visseri* (figs. 1, 2 and 3). These analyses recovered all 16 major gene lineages clustering with all homologs known from the model species *Arabidopsis thaliana.* We identified orthologs of all critical floral meristem and floral organ identity genes (as defined by [21, 67, 83, 90] including *AG*, *AGL6*, *AP3*, *AP1*/*FUL*, *PI*, *SEP1/2/4*, and *SEP3* (Fig. 3; suppl. Fig S1–S5) in all taxa from which a complete floral transcriptome was generated (Supplementary Tables S2, S5)*.* The only exception is the absence of a *SEP3* ortholog expressed in sampled transcriptomes of* H. visseri* (Fig. 3; suppl. Fig. S1). Other MADS-box genes involved in ovule formation, seed pigmentation, and fruit development, including *STK* and *ABS* (as defined by [3, 22, 60, 70]), were found in both parasitic and non-parasitic Piperales (Fig. 3; suppl. Figs. S2, S3). Interestingly, the *TDR8* homologs, which are known for their role in stamen, carpel, and fruit development in tomato (as defined by [23]), were found only in species of *Aristolochia* and *Thottea* (Fig. 3, Suppl. Fig. S3). Most MIKC^c^ MADS-box genes involved in reproductive transition (i.e., the shift from shoot apical meristems to reproductive meristems) were isolated from non-parasitic Piperales. These include the flowering activators *AGL16/17*, *AGL24*, and *SOC1* (as defined by [32, 47, 48]), and the repressors *AGL15/18* and *SVP* (as defined by [1, 34]). From all flowering transition MADS-box genes, only *SOC1* homologs were present in *H. visseri* (Fig. 3, Suppl. Fig. S4). Finally, infrequently found MADS-box genes include *FLC* homologs, in *Thottea* only*,* and *AGL12/XAANTAL* homologs, restricted to *Aristolochia*, *Piper*, and *Thottea* (Fig. 3, Suppl. Figs. S1, S3)*. FLC* is a key flowering repressor in species that require vernalization (as defined by [56]). Conversely, *AGL12/XAANTAL* homologs in *Arabidopsis* have been associated with root growth and function as flowering transition activators (as defined by [84]).

*Hydnora visseri* MADS-box genes often appeared as sister to those of *Aristolochia* and *Thottea* (Fig. 3; Suppl. Figs. S1-S5). This is true for both nucleotide and amino acid-based alignments. However, some genes (e.g., *HyviAP3* and *HyviFUL*) appeared as sister to those of all other perianth-bearing Piperales or occasionally clustered with *Nymphaea* or *Amborella* sequences.

**MADS-box gene evolution across Piperales.** With the goal of understanding the evolution of all MADS-box genes across Piperales, we mapped large-scale and local gene duplications. We defined large-scale duplications as those involving more than one genus in the order or within the genus *Aristolochia*, whereas local duplications correspond to those restricted to a single species. Considering that data come from transcriptomes only, we cannot assess whether local gene duplications are true paralogs or if they correspond to alternative splicing events of a single gene. However, the first scenario is more plausible considering that all local duplicates in the phylogenetic reconstructions of the outgroup *Nymphaea thermarum,* (verified at a genome-scale of this species) are reported as true paralogs.

***A-class and E-class genes. SEPALLATA*** (*SEP*) genes are required for the proper functioning of A, B, and C-class genes (as reported by [24, 68]). Additionally, *SEP3* has been identified as the most critical component of tetrameric complexes physically interacting with other floral organ identity MADS-box genes [40, 55]. *SEP* genes have diversified into two large clades in angiosperms, namely *SEP1/2/4* and *SEP3* [93]. Both gene lineages were recovered in our analyses (Suppl. Fig. S1). *SEP1/2/4* homologs were retained primarily as single copies with a large-scale duplication prior to the diversification of Piperaceae and Saururaceae (UFBS = 99) and another in *Aristolochia* subg*. Pararistolochia* (UFBS = 100) (Suppl. Fig. S1). Local *SEP1/2/4* duplications were identified in *Houttuynia cordata* Thunb., *Piper nigrum* L., and *Thottea siliquosa.* On the other hand, two duplications within *SEP3* homologs were recovered. The first one (UFBS = 74) preceded the diversification of Piperales. The second duplication coincides with the diversification of *Aristolochia* subg*. Pararistolochia* (UFBS = 100). Species-level duplications of *SEP3* homologs were detected only in *P. nigrum*.

Perianth identity candidate genes include *AP1* in *Arabidopsis thaliana* (Coen and Meyerowitz 1990) and *AGL6* in *Nigella damascena* [89]. These transcription factors belong to two different gene lineages, the *AP1/FUL* and the *AGL6*, respectively. In Piperales, the *AP1/FUL* genes duplicated prior to the diversification of Piperaceae, with two copies retained in *Piper* and *Peperomia* (UFBS = 99; Suppl. Fig. S1). They also duplicated prior to the diversification of *Asarum* and *Saruma* (UFBS = 100), followed by the loss of one copy in *Asarum* (Suppl. Fig. S1). *AP1/FUL* genes also duplicated in *Aristolochia* subg*. Pararistolochia* (UFBS = 95; Suppl. Fig. S1). Local duplications of *AP1/FUL* genes occur in *Aristolochia salvadorensis*, *Piper methysticum* G.Forst., *P. nigrum* and *Thottea siliquosa*. Conversely, *AGL6* genes underwent a duplication prior to the diversification of all members of perianth-bearing Piperales (UFBS = 92; Suppl. Fig. S1), with one copy conserved in all genera, and the other copy retained only in *Asarum* and *Saruma*. An additional large-scale duplication occurred independently prior to the diversification of perianth-less Piperales (UFBS = 82; Suppl. Fig. S1). Local duplications of *AGL6* genes occur in *P. nigrum* and *Thottea siliquosa*.

***B-class and B-sister genes.*** The *AP3* and *PI* gene lineages, known to be crucial for petal and stamen identity, are mostly found as single-copy genes across Piperales (Suppl. Fig. S2). However, there are two exceptions: an *AP3* gene duplication prior to the diversification of Piperaceae and Saururaceae (UFBS = 99), and an additional duplication of the same gene lineage in the species of *Aristolochia* subg*. Siphisia* (UFBS = 95). Large-scale duplications of *PI* genes were only detected prior to the diversification of Piperaceae and Saururaceae (UFBS = 97). Local duplications of *PI* were found in *Houttuynia cordata* and *Thottea siliquosa*. The B-sister *ABS/GOA* genes, linked to seed pigmentation and fruit development, were also found predominantly as single-copy in Piperales, and local *ABS* duplications were found only in *Aristolochia deltantha* (Suppl. Fig. S2). Interestingly, *ABS* homologs were not isolated from several species of *Aristolochia*, more likely due to the limited sampling of late seed/fruit developmental stages in the reference transcriptomes.

***C and D-class genes.*** The homologs of *AG*, the canonical stamen and carpel organ identity gene in *Arabidopsis*, and homologs of *STK*, essential for ovule development, were found as single-copy genes in most Piperales (Suppl. Fig. S3). However, two large-scale duplications of *AG* were detected, one before the diversification of all Piperales (UFBS = 51), and another prior to the diversification of *Asarum* and *Saruma* (UFBS = 68; Suppl. Fig. S3).

***MADS-box genes involved in fruit development. TDR8*** has been linked to proper carpel-to-fruit transition and fruit maturation in tomato. *TDR8* genes were detected only in *Aristolochia* and *Thottea* (Suppl. Fig. S3). The phylogenetic reconstruction performed here suggests an early duplication in *Aristolochia* (UFBS = 100). The two copies were retained only in the species of *Aristolochia* subg*. Siphisia,* whereas all other members of *Aristolochia* have one copy*.* An additional local duplication was found in *Aristolochia* subg*. Pararistolochia* (UFBS = 100)*.* Local *TDR8* duplicates were found in *A. lindneri*, *A. manshuriensis,* and *T. siliquosa.*

***MADS-box genes involved in reproductive transition.*** Canonical flowering activators include *AGL16/17* and *SOC1.* Interestingly, *AGL16*/*17* homologs were hardly isolated, and homologs were only found in *Aristolochia arborea* and *A. clematitis* (Suppl. Fig. S5). In contrast, *SOC1* genes were duplicated twice (both UFBS = 95) before the diversification of *Asarum* and *Saruma*, and triplicated in *Aristolochia* subg*. Siphisia* (UFBS = 100; Suppl. Fig. S4). An additional large-scale duplication was mapped for all perianth-less Piperales (UFBS = 85; Suppl. Fig. S4). Local *SOC1* duplicates were found in *Asarum canadense*, *Aristolochia arborea*, *A. macrophylla*, *A. manshuriensis*, *A. praevenosa, Piper nigrum*, and *Thottea siliquosa*.

Flowering repressors *AGL15/18, FLC*, and *SVP* have different evolutionary histories. *FLC* genes were found only in *Thottea siliquosa*, with four local duplicates, in *Piper nigrum* and *Saururus chinensis* (Suppl. Fig. S1). *AGL15/18* were detected as single-copy genes in all sampled species, with the exception of local duplications in *Aristolochia deltantha*, *Asarum canadense*, and *Saruma henryi* (Suppl. Fig. S5). Finally, *AGL24*/*SVP* homologs were found triplicated in members of *Aristolochia* subg*. Siphisia* (UFBS = 100; Suppl. fig. S8)*.* Local *AGL24*/*SVP* duplications were found in *Aristolochia arborea*, *A. praevenosa, Piper nigrum*, and *Thottea siliquosa*.

**Fig. 4:**
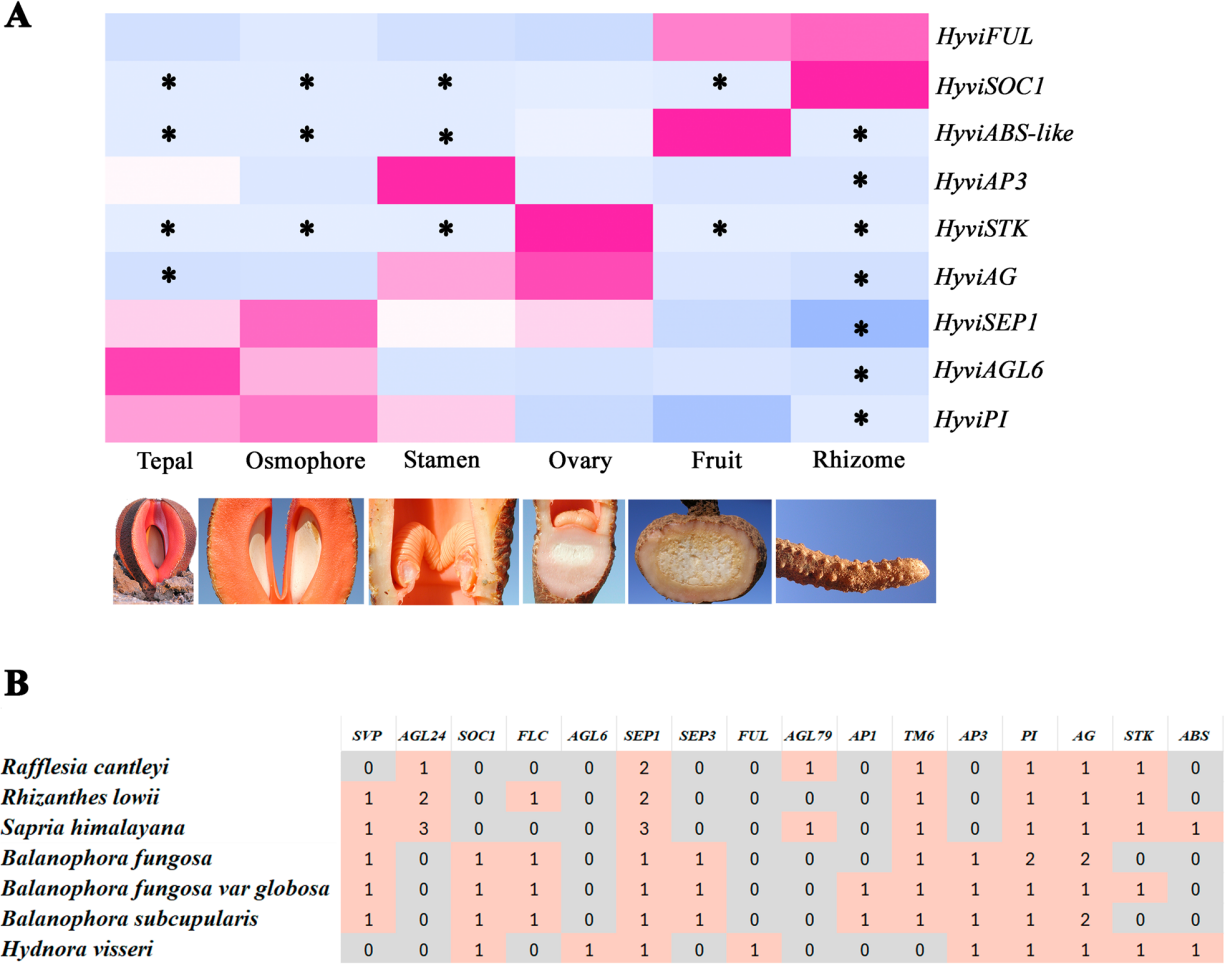
**A**. Heatmap showing selected MADS-box gene expression in different plant organs of *Hydnora visseri*. Gene expression levels are pointed at the top of the heatmap, with pink and blue indicating up-regulated and down-regulated expression, respectively. **B**. Summary table of MADS-box genes and copy numbers recovered in selected parasitic species (modified from [13, 25, 63])

***Expression of the MADS-box genes found in Hydnora visseri.*** In order to better understand the putative roles of MADS-box genes identified in *H. visseri*, we assessed their expression patterns in the transcriptomes. The data show normalized TPM values with respect to the *ACTIN* content for each gene in all six reference transcriptomes belonging to the rhizome and the dissected floral organs, viz. tepals, osmophores, stamens, gynoecium with ovules, and fruits with seeds (Fig. 4).

*HyviSOC1* and *HyviFUL* are the only two MADS-box genes expressed in the rhizome. Expression of *HyviSEP* was detected broadly in all floral parts and in fruits. *HyviAGL6* and *HyviPI* were more highly transcribed in the tepals and the osmophore. *HyviPI* and *HyviAP3* showed overlapping expression in the stamens, where they reached their highest expression levels. *HyviAG* and *HyviSTK* were found more strongly expressed in the gynoecium with forming ovules. Finally, *HyviABS-like* was more strongly expressed in fruits (Fig. 4).

**Fig. 5:**
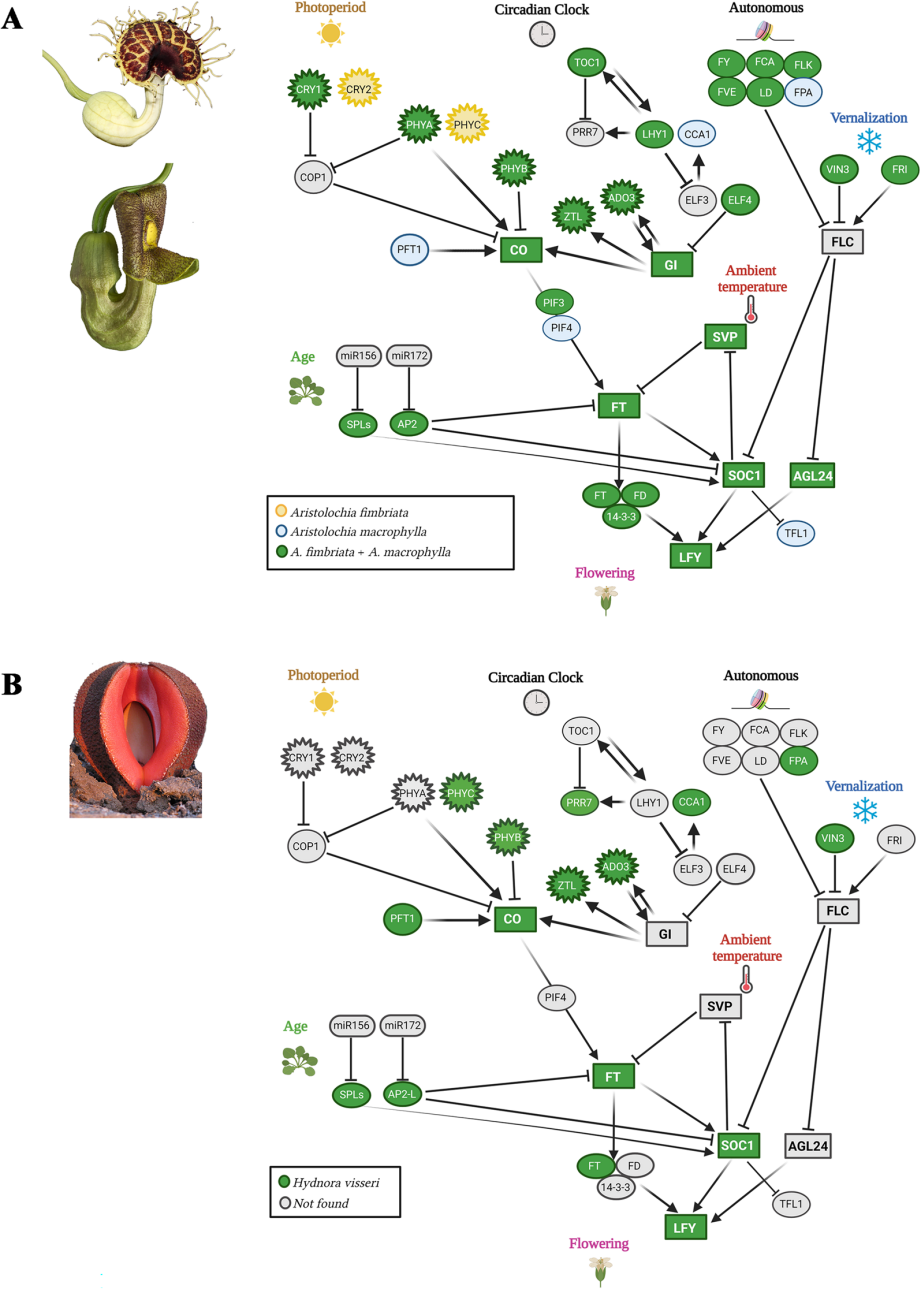
Summarized photoperiod, circadian clock, autonomous, and vernalization pathways (following [49]) that control flowering in *Aristolochia* species (**A**) and in the holoparasitic *Hydnora visseri* (**B**)*.* Yellow-colored genes in **A** were found only in *A. fimbriata*; blue-colored genes were only found in *A. macrophylla*; green-colored genes were found in both species. Green-colored genes in **B** are endogenous in *H. visseri*; gray-colored genes (expected from the canonical pathways) are lacking in *Hydnora*

***Tracking the retention of the flowering regulators in Hydnora visseri. *** To assess whether *H. visseri* relies on its host for the flowering transition, or if it has autologous signals to induce flowering, we annotated all expressed major flowering regulators in the photoperiod, circadian clock, and autonomous and vernalization pathways (Fig. 5). For comparison, we annotated the same expressed genes in two species of *Aristolochia* species, namely, *A. fimbriata* and *A. macrophylla* (Fig. 5). Major differences were found in the annotation of the autonomous pathway genes, most of which could not be isolated from the *H. visseri* transcriptome, including *FLOWERING LOCUS Y* (*FY*), *FLOWERING CONTROL LOCUS A* (*FCA*), *FLOWERING LOCUS K* (*FLK*), *FLOWERING LOCUS VE* (*FVE*) and *LUMINIDEPENDENS* (*LD*). Notably, critical factors in the flowering transition, such as *GIGANTEA* (*GI*), *PHYTOCHROME INTERACTING FACTOR 3/4* (*PIF3/4*), and *AGL24/SVP* were missing in the *H. visseri* transcriptome (Fig. 5) as well as major components of the circadian clock pathway, such as the *TIMING OF CAB1* (*TOC1*) gene. However, the *PSEUDO-RESPONSE REGULATOR 7* (*PRR7*) genes were identified (Suppl. fig. S6). Conversely, the transcriptome contained upstream factors in the photoperiod like the three *PHYTOCHROMES* (*PHYA*, *PHYB*, *PHYC*) and *CONSTANS* (*CO*) that were confirmed to belong to *Hydnora* by similarity and phylogenetic analyses (Fig. 5; Suppl. Figs. S7, S8). All other key flowering integrators, including *ADAGIO PROTEIN3* (*ADO3*), *CIRCADIAN CLOCK ASSOCIATED 1* (*CCA1*), *FLOWERING LOCUS T* (*FT*), *SQUAMOSA-PROMOTER BINDING PROTEIN-LIKE* (*SPLs*), *ZEITLUPE* (*ZTL*), *AP2-*related genes, *SOC1*, and *LEAFY* (*LFY*), were expressed in *H. visseri*.

## Discussion

***Only critical flowering integrators are active in Hydnora visseri.*** Our results suggest that holoparasitism in *Hydnora* is accompanied by an expression reduction of the endogenous flowering signals, in particular those from the autonomous pathway and the circadian clock control. In comparison to the transcriptomes derived from *Aristolochia* species, which are all autotrophs, we did not find any contigs in the *H. visseri* transcriptomes linked to the maintenance of circadian rhythms, such as the critical morning expressed factor *LATE ELONGATED HYPOCOTYL* (*LHY*) or the evening-phased factor *GIGANTEA* (*GI*). These genes play crucial roles as flowering integrators in other plants [10]. Downstream circadian clock integrators like *EARLY FLOWERING 4* (*ELF4*) were not found in *H. visseri* transcriptomes*,* suggesting that the circadian clock route is not as active. Specifically, the only two circadian clock regulators expressed in *H. visseri* are the *MYB* gene *CIRCADIAN CLOCK ASSOCIATED 1* (*CCA1*) and the day-phased transcriptional regulator *PSEUDORESPONSE REGULATOR 7* (*PRR7*)*,* which is an essential component of the temperature-sensitive circadian clock. It is unclear whether *CCA1* and *LHY* are core-eudicot or Brassicaceae specific duplicates. Thus, it is likely that early diverging angiosperms, including the order Piperales, only have pre-duplication genes, in this case more similar to *CCA1* [50].

A similar scenario stands out when comparing the autonomous route for flowering of *Aristolochia* spp. and *H. visseri*. Out of the six major controllers (*FLOWERING CONTROL LOCUS A* (*FCA*), *FLOWERING LOCUS K* (*FLK*), *FLOWERING LOCUS PA* (*FPA*), *FLOWERING LOCUS VE* (*FVE*), *FLOWERING LOCUS Y* (*FY*), and *LUMINIDEPENDENS* (*LD*)), only *FPA* was found in *H. visseri.* The role of the autonomous flowering pathway is to accelerate flowering, regardless of day length, by inhibiting the central flowering repressor *FLOWERING LOCUS C* (*FLC*), both through RNA regulation as well as through chromatin modifications [15]. It is uncertain what the target genes are, in the absence of expression of *FLC* homologs in most perianth-bearing Piperales; yet, it is evident that autonomous genes are mostly lacking in *Hydnora*. When compared to other distantly related holoparasitic angiosperms, such as *Balanophora fungosa* J.R.Forst. & G.Forst., *B. subcupularis* Tam (Balanophoraceae), and *Sapria himalayana* Griff. (Rafflesiaceae), the same trend of reduction in circadian clock genes is detected [13, 25].

The most complete route in the *Hydnora visseri* is the one activated in response to light stimuli. Upstream responsive phytochromes (*PHYA*, *PHYB* and *PHYC*) as well as the downstream central regulators (including the *PHYTOCHROME AND FLOWERING TIME REGULATORY PROTEIN* (*PFT1*), *CONSTANS* (*CO*), *FLOWERING LOCUS T* (*FT*), *SUPRESSOR OF CONSTANS 1* (*SOC1*) and *LEAFY* (*LFY*)) are endogenous and active. This strongly suggests that photoperiod-induced flowering is intact in *Hydnora* and it does not depend upon host flowering signals, unlike what has been shown to occur in *Cuscuta,* which seems to sequester the host FT mobile signals for its own flowering [96]. The photoperiod-induced flowering is also largely retained in *Balanophora fungosa, B. subcupularis*, and *Sapria himalayana*, with few differences. Whereas *FLC* has been detected only in *Balanophora* species [13, 25], *CO* has been found only in *H. visseri*. The retention of *CO* in *Hydnora* may reflect its adaptation to arid and heavily light-exposed environments, where photoperiodic cues are likely essential, in contrast to the shaded understory habitats occupied by *Balanophora* and *Sapria*. Interestingly, both underground organs (i.e., the tuber in *Balanophora* and the rhizome in *Hydnora*) express *SOC1*, which has been proposed as a critical integrator of flowering time signals and a key factor for targeting downstream floral organ identity genes [48, 97]. In turn, *SOC1* could be the most conserved autonomous hub (i.e., critical flowering integrator) found in all holoparasites having modified stems studied so far [25], including in the first emergence of holoparasitism in angiosperm evolution, represented here by *Hydnora*. Like in *Balanophora* and *Sapria*, all other MADS box genes important for root development and the reproductive transition, like *AGL12*, *AGL15*, *AGL16*, and *AGL17* (see [13, 25]), are not expressed in *Hydnora*, suggesting that these transcription factors are no longer essential in these species.

It is important to highlight that our study also revealed that *SOC1* genes underwent five large-scale duplication events in temperate taxa within the perianth-bearing Piperales, i.e., the Asaraceae, and members of *Aristolochia* subg*. Siphisia*, which are seasonally exposed to extreme temperatures and day length variations. As *SOC1* copies have shown diverse responses to photoperiod in other species [52], it is likely that the numerous homologs of such critical floral integrators in Aristolochiaceae and Asaraceae have effects in flowering responses.

**Hydnora visseri floral organ identity genetic toolkit resembles that of Aristolochia. Hydnora** is known as one of the strangest plants in the world [58, 85]. However, its floral groundplan and underlying MADS-box genes are not too dissimilar from those of *Aristolochia* [71]*. Hydnora* has a complete set of floral organ identity MADS-box genes, perhaps with the exception of *SEPALLATA*, as *Hydnora* lacks an ortholog of *SEP3* but retains the *SEP1/2/4* copies. This is unusual, as homologs in the *SEP3* clade are the most critical factors for floral organ identity tetrameric complex formation [40]. Moreover, the *SEP3* ortholog is the copy with a stronger expression and larger set of interactions in *Aristolochia* [69, 82]. It is possible that the *SEP1/2/4* homolog is acting in tetrameric complexes instead, which points to a larger degree of redundancy than expected between the two *SEP* copies present in early diverging angiosperms. All other MADS-box genes closely resemble the expression patterns recorded for floral organ identity genes in *Aristolochia*. For instance, the uniseriate perianth of *Hydnora* consists of three thickened osmophore-bearing organs that express *SEP* and *AGL6*, the same gene classes that are hypothesized to control sepal identity in *A. fimbriata* [67, 69]. Our studies have shown that osmophores structurally and developmentally correspond to specialized regions of the inner surface of the perianth in both taxa, and that they do not represent additional floral organs. The fact that tepals in *Hydnora* most likely correspond to sepals was also noted by Vaccaneo [86] based on their position opposite to stamens. Late expression of *PI* in the perianth can occur linked to the late petaloid features of the three sepals in both *Aristolochia* and *Hydnora* (Suppl. fig. S2; [41, 67]). Stamen identity in *Hydnora* can be regulated by the expression of *AP3*, *PI*, *AG*, and *SEP*, while carpel identity seems to require the activity of *AG*, *STK,* and *SEP,* the same genes that control stamen and carpel identity in *Aristolochia* [67, 82]. Even the expression of *FUL* and *ABS-like* genes in fruits seems to be concordant to the more critical roles of these transcription factors during seed and fruit development [70], Pabón-Mora et al. [60]; [26]. The only unexpected result is the absence of *STK* expression during the carpel-to-fruit transition in *Hydnora*, as *STK* is expressed in both the capsule dehiscence zone and in the seeds of *A. fimbriata* [82]. However, low expression of *STK* in *Hydnora* fruits may be linked to the fact that they lack a defined dehiscence zone, as they open through irregular slits.

Hydnoraceae represents the earliest known emergence of holoparasitism in angiosperm evolution. Therefore, comparing the floral genes retained in *Hydnora* with those in other holoparasitic plants may help illuminate core floral modules that have persisted over evolutionary time. Similar floral organ identity genetic toolkits have been reported in the distantly related holoparasites *Balanophora* (Balanophoraceae; [25]) and *Sapria* (Rafflesiaceae; [13]) pointing to critical functions of MADS-box genes in plant reproduction via the identity and elaboration of floral organs. However, in contrast to other holoparasitic plants, *Hydnora* might be one of the angiosperms with the simplest genetic developmental toolkit. This is likely supported by its phylogenetic position as a member of the magnoliids, since many MADS-box gene lineages, including *APETALA1/FRUITFULL* (*AP1/FUL*), *APETALA3/TOMATO MADS 6* (*AP3/TM6*), *AGAMOUS* (*AG*), duplicated concomitantly with the diversification of monocots and core eudicots [45, 46, 51] and became evolutionarily redundant only after the diversification of Piperales. Assessing the evolution of the active MADS-box genes in *Hydnora* may be of great interest, given that the processes that they control are among the most critical for plant survival. Also, this minimal genetic toolkit may be worth exploring in more detail in *Prosopanche*, the second holoparasitic genus in the family, as well as in the successive non-parasitic sister lineages.

***Remarks on perianth evolution across autotrophic related lineages.*** In-depth analyses of floral organ identity MADS-box genes in perianth-bearing Piperales allowed us to identify a pivotal cue regarding perianth identity genes. Both the *AGL6* and the *SEP3* orthologs were duplicated prior to the diversification of the perianth-bearing lineage, and subsequently the two resulting copies were retained only in Asaraceae (Fig. 4). The genera *Aristolochia, Hydnora*, and *Thottea* (and most likely *Prosopanche* and *Lactoris*) have lost one of the two copies in both gene lineages. This observation also coincides with the presence of a biseriate perianth in *Saruma*, and the occasional presence of vestigial petals in some species of *Asarum* [29, 66], in contrast to the uniseriate perianth in the remaining genera. Considering that no other floral identity MADS-box gene lineages show such duplication pattern with subsequent losses in Hydnoraceae and Aristolochiaceae, it is worth performing additional studies on the expression and protein–protein interactions of the perianth identity candidate genes in a comparative manner across all Piperales families including Piperaceae and Saururaceae. Conversely, the broadly expressed *FUL* genes, with pleiotropic roles in floral meristem, sepal and petal identity, and fruit development, duplicated locally prior to the diversification of *Asarum* and *Saruma*, resulting in two recent paralogs restricted only to these two genera (Suppl. Fig. S1). As gene duplication can promote and enhance functional evolution, follow-up studies are needed to assess the contribution of all MADS-box genes found duplicated in these two genera, and to better understand functional changes that might have shaped the extraordinary floral variation across the order Piperales.

## Supplementary Information

Additional file 1.

Additional file 2 (Supplementary Figure 1. Phylogenetic reconstruction of AGAMOUS-LIKE 6 (AGL6), SEPALLATA (SEP), APETALA1/FRUITFUL (FUL), and FLOWERING LOCUS C (FLC) gene subclades; each gene lineage is differentially colored and labeled; yellow stars point to large-scale duplication events; gray stars point to local or species-specific duplications; red arrowheads point to MADS-box homologs detected in the Hydnora visseri transcriptome. Supplementary Figure 2. Phylogenetic reconstruction of the APETALA 3 (AP3), PISTILLATA (PI) and ARABIDOPSIS B-SISTER (ABS) gene subclades; each gene lineage is differentially colored and labeled; yellow stars point to large-scale duplication events; gray stars point to local or species-specific duplications; red arrowheads point to MADS-box homologs detected in the Hydnora visseri transcriptome. Supplementary Figure 3. Phylogenetic reconstruction of the SEEDSTICK (STK), AGAMOUS (AG), AGAMOUS-like 12 (AGL12), and TDR8 gene subclades; each gene lineage is colored and labeled; yellow stars point to large-scale duplication events; gray stars point to local or species-specific duplications; red arrows point to MADS-box homologs detected in the Hydnora visseri transcriptome. Supplementary Figure 4. Phylogenetic reconstruction of the SUPRESSOR OF CONSTANS (SOC1) gene subclade; each gene lineage is colored and labeled; yellow stars point to large-scale duplication events; gray stars point to local or species-specific duplications; red arrowhead points to MADS-box homologs detected in the Hydnora visseri transcriptome. Supplementary Figure 5. Phylogenetic reconstruction of the AGAMOUS-LIKE 15 (AGL15), AGL16 and SHORT VEGETATIVE PHASE (SVP) gene subclades; each gene lineage is colored and labeled; yellow stars point to large-scale duplication events; gray stars point to local or species-specific duplications. Supplementary Figure 6. Phylogenetic reconstruction of the TIMING OF CAB 1 (TOC1) and PSEUDO-RESPONSE REGULATOR 7 circadian clock genes. Arrow points to H. visseri homologs; arrowheads point to canonical Arabidopsis genes. Supplementary Figure 7. Phylogenetic reconstruction of the PHYTOCHROME A, B and C genes. Arrow points to H. visseri homologs; arrowheads point to canonical Arabidopsis genes. Supplementary Figure 8. Phylogenetic reconstruction of the CONSTANS and CONSTANS-like genes. Arrow points to H. visseri homologs; arrowheads point to canonical Arabidopsis genes.)

## Data Availability

Hydnora transcriptomes are deposited in NCBI under bio-accessions PRJNA1111920, SAMN41406291: Hyd-Fruit, SAMN41406292: Hyd-Gynoecium, SAMN41406293: Hyd-Osmophore, SAMN41406294: Hyd-Rhizome, SAMN41406295: Hyd-Stamens, SAMN41406296: Hyd-Tepals. Also, all 246 genes from the gene tree analysis have been submitted to GenBank.
