# Supplementary material for: The floral ABCs of Hydnora, one of the most bizarre parasitic plants in the world, and its autotrophic relatives of the order Piperales
Source: EvoDevo. 2025 Oct 2;16:16. doi: 10.1186/s13227-025-00252-8 (PMC12490054; doi:10.1186/s13227-025-00252-8)
Supplement: Supplementary file 2 — Additional file 2 (Supplementary Figure 1. Phylogenetic reconstruction of AGAMOUS-LIKE 6 (AGL6), SEPALLATA (SEP), APETALA1/FRUITFUL (FUL), and FLOWERING LOCUS C (FLC) gene subclades; each gene lineage is differentially colored and labeled; yellow stars point to large-scale duplication events; gray stars point to local or species-specific duplications; red arrowheads point to MADS-box homologs detected in the Hydnora visseri transcriptome. Supplementary Figure 2. Phylogenetic reconstruction of the APETALA 3 (AP3), PISTILLATA (PI) and ARABIDOPSIS B-SISTER (ABS) gene subclades; each gene lineage is differentially colored and labeled; yellow stars point to large-scale duplication events; gray stars point to local or species-specific duplications; red arrowheads point to MADS-box homologs detected in the Hydnora visseri transcriptome. Supplementary Figure 3. Phylogenetic reconstruction of the SEEDSTICK (STK), AGAMOUS (AG), AGAMOUS-like 12 (AGL12), and TDR8 gene subclades; each gene lineage is colored and labeled; yellow stars point to large-scale duplication events; gray stars point to local or species-specific duplications; red arrows point to MADS-box homologs detected in the Hydnora visseri transcriptome. Supplementary Figure 4. Phylogenetic reconstruction of the SUPRESSOR OF CONSTANS (SOC1) gene subclade; each gene lineage is colored and labeled; yellow stars point to large-scale duplication events; gray stars point to local or species-specific duplications; red arrowhead points to MADS-box homologs detected in the Hydnora visseri transcriptome. Supplementary Figure 5. Phylogenetic reconstruction of the AGAMOUS-LIKE 15 (AGL15), AGL16 and SHORT VEGETATIVE PHASE (SVP) gene subclades; each gene lineage is colored and labeled; yellow stars point to large-scale duplication events; gray stars point to local or species-specific duplications. Supplementary Figure 6. Phylogenetic reconstruction of the TIMING OF CAB 1 (TOC1) and PSEUDO-RESPONSE REGULATOR 7 circadian clock gene [file 13227_2025_252_MOESM2_ESM.pdf]

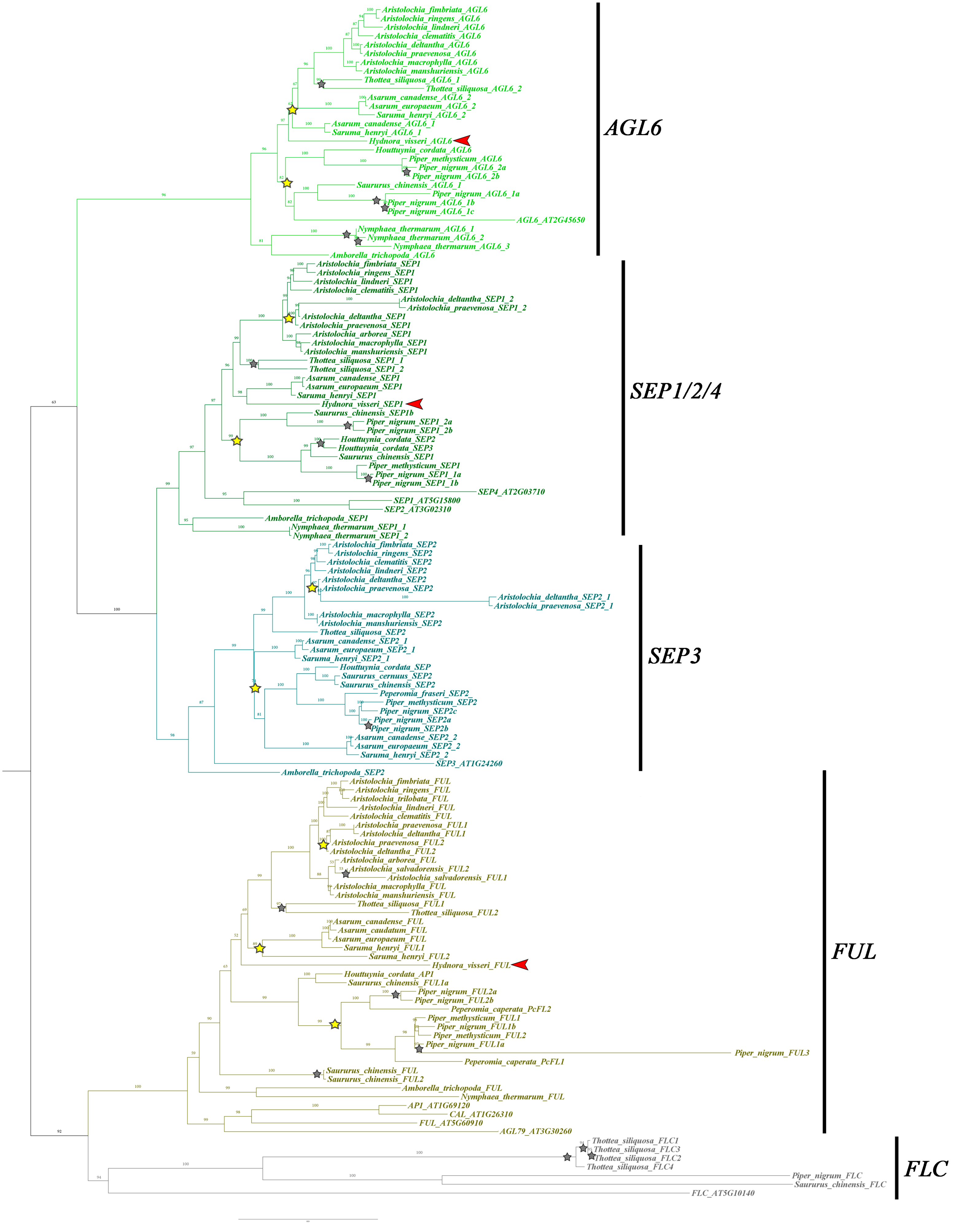
AGL6
Aristolochia_fimbriata_AGL6
Aristolochia_ringens_AGL6
Aristolochia_lindneri_AGL6
Aristolochia_clematitis_AGL6
Aristolochia_deltantha_AGL6
Aristolochia_praevenosa_AGL6
Aristolochia_macrophylla_AGL6
Aristolochia_manshuriensis_AGL6
Thottea_siliquosa_AGL6_1
Thottea_siliquosa_AGL6_2
Asarum_canadense_AGL6_2
Asarum_europaeum_AGL6_2
Saruma_henryi_AGL6_2
Asarum_canadense_AGL6_1
Saruma_henryi_AGL6_1
Hydnora_visseri_AGL6
Houttuynia_cordata_AGL6
Piper_methysticum_AGL6
Piper_nigrum_AGL6_2a
Piper_nigrum_AGL6_2b
Saururus_chinensis_AGL6_1
Piper_nigrum_AGL6_1a
Piper_nigrum_AGL6_1b
Piper_nigrum_AGL6_1c
AGL6_AT2G45650
Nymphaea_thermarum_AGL6_1
Nymphaea_thermarum_AGL6_2
Nymphaea_thermarum_AGL6_3
Amborella_trichopoda_AGL6
SEP1/2/4
Aristolochia_fimbriata_SEP1
Aristolochia_ringens_SEP1
Aristolochia_lindneri_SEP1
Aristolochia_clematitis_SEP1
Aristolochia_deltantha_SEP1_2
Aristolochia_praevenosa_SEP1_2
Aristolochia_deltantha_SEP1
Aristolochia_praevenosa_SEP1
Aristolochia_arborea_SEP1
Aristolochia_macrophylla_SEP1
Aristolochia_manshuriensis_SEP1
Thottea_siliquosa_SEP1_1
Thottea_siliquosa_SEP1_2
Asarum_canadense_SEP1
Asarum_europaeum_SEP1
Saruma_henryi_SEP1
Hydnora_visseri_SEP1
Saururus_chinensis_SEP1b
Piper_nigrum_SEP1_2a
Piper_nigrum_SEP1_2b
Houttuynia_cordata_SEP2
Houttuynia_cordata_SEP3
Saururus_chinensis_SEP1
Piper_methysticum_SEP1
Piper_nigrum_SEP1_1a
Piper_nigrum_SEP1_1b
SEP4_AT2G03710
SEP1_AT5G15800
SEP2_AT3G02310
Amborella_trichopoda_SEP1
Nymphaea_thermarum_SEP1_1
Nymphaea_thermarum_SEP1_2
SEP3
Aristolochia_fimbriata_SEP2
Aristolochia_ringens_SEP2
Aristolochia_clematitis_SEP2
Aristolochia_lindneri_SEP2
Aristolochia_deltantha_SEP2
Aristolochia_praevenosa_SEP2
Aristolochia_deltantha_SEP2_1
Aristolochia_praevenosa_SEP2_1
Aristolochia_macrophylla_SEP2
Aristolochia_manshuriensis_SEP2
Thottea_siliquosa_SEP2
Asarum_canadense_SEP2_1
Asarum_europaeum_SEP2_1
Saruma_henryi_SEP2_1
Houttuynia_cordata_SEP
Saururus_cernuus_SEP2
Saururus_chinensis_SEP2
Peperomia_fraseri_SEP2
Piper_methysticum_SEP2
Piper_nigrum_SEP2c
Piper_nigrum_SEP2a
Piper_nigrum_SEP2b
Asarum_canadense_SEP2_2
Asarum_europaeum_SEP2_2
Saruma_henryi_SEP2_2
SEP3_AT1G24260
Amborella_trichopoda_SEP2
FUL
Aristolochia_fimbriata_FUL
Aristolochia_ringens_FUL
Aristolochia_trilobata_FUL
Aristolochia_lindneri_FUL
Aristolochia_clematitis_FUL
Aristolochia_praevenosa_FUL1
Aristolochia_deltantha_FUL1
Aristolochia_praevenosa_FUL2
Aristolochia_deltantha_FUL2
Aristolochia_arborea_FUL
Aristolochia_salvadorensis_FUL2
Aristolochia_salvadorensis_FUL1
Aristolochia_macrophylla_FUL
Aristolochia_manshuriensis_FUL
Thottea_siliquosa_FUL1
Thottea_siliquosa_FUL2
Asarum_canadense_FUL
Asarum_caudatum_FUL
Asarum_europaeum_FUL
Saruma_henryi_FUL1
Saruma_henryi_FUL2
Hydnora_visseri_FUL
Houttuynia_cordata_AP1
Saururus_chinensis_FUL1a
Piper_nigrum_FUL2a
Piper_nigrum_FUL2b
Peperomia_caperata_PcFL2
Piper_methysticum_FUL1
Piper_nigrum_FUL1b
Piper_methysticum_FUL2
Piper_nigrum_FUL1a
Piper_nigrum_FUL3
Peperomia_caperata_PcFL1
Saururus_chinensis_FUL
Saururus_chinensis_FUL2
Amborella_trichopoda_FUL
Nymphaea_thermarum_FUL
AP1_AT1G69120
CAL_AT1G26310
FUL_AT5G60910
AGL79_AT3G30260
FLC
Thottea_siliquosa_FLC1
Thottea_siliquosa_FLC3
Thottea_siliquosa_FLC2
Thottea_siliquosa_FLC4
Piper_nigrum_FLC
Saururus_chinensis_FLC
FLC_AT5G10140

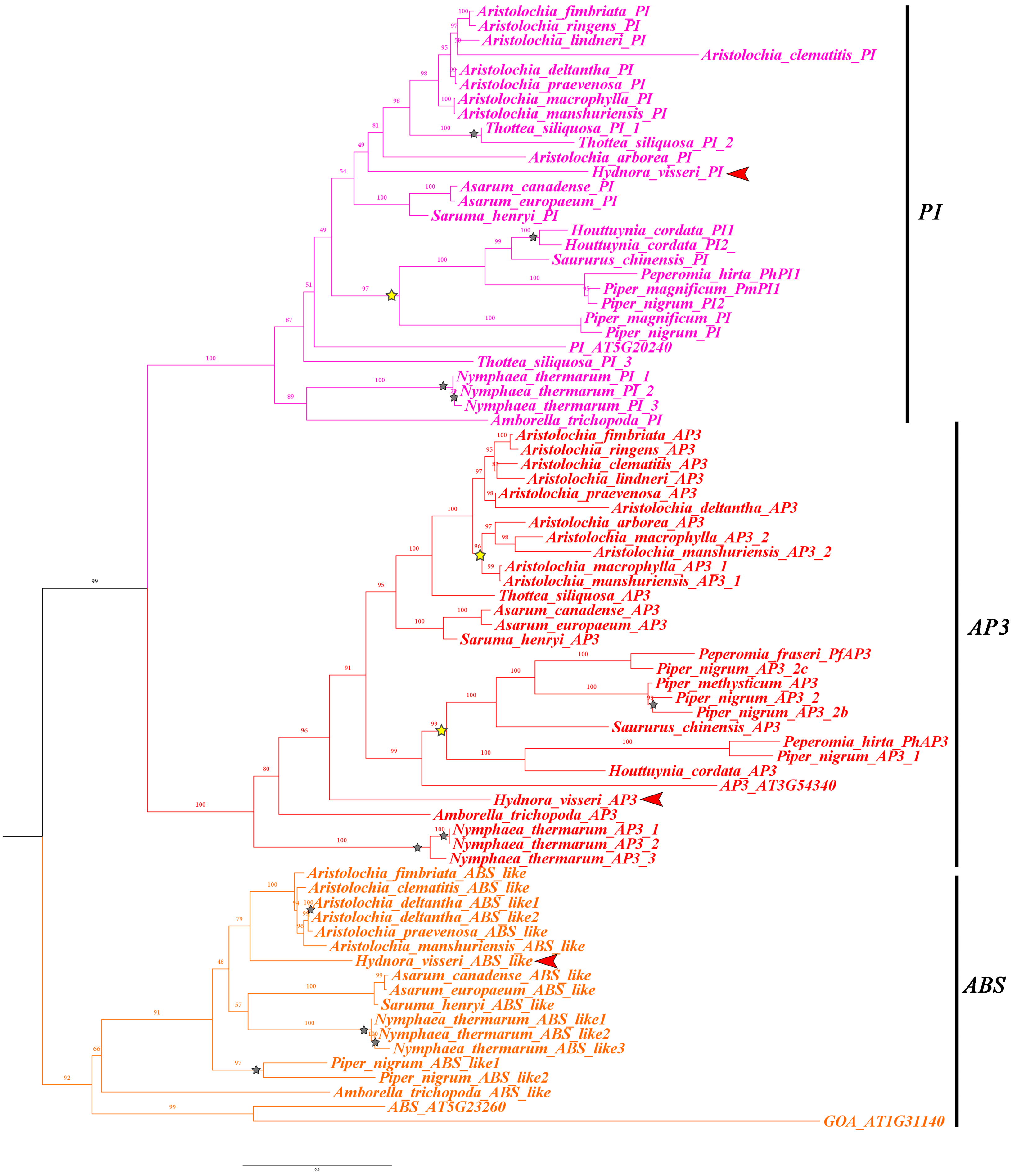
Aristolochia_fimbriata_PI
Aristolochia_ringens_PI
Aristolochia_lindneri_PI
Aristolochia_clematitis_PI
Aristolochia_deltantha_PI
Aristolochia_praevenosa_PI
Aristolochia_macrophylla_PI
Aristolochia_manshuriensis_PI
Thottea_siliquosa_PI_1
Thottea_siliquosa_PI_2
Aristolochia_arborea_PI
Hydnora_visseri_PI
Asarum_canadense_PI
Asarum_europaeum_PI
Saruma_henryi_PI
Houttuynia_cordata_PI1
Houttuynia_cordata_PI2_
Saururus_chinensis_PI
Peperomia_hirta_PhPI1
Piper_magnificum_PmPI1
Piper_nigrum_PI2
Piper_magnificum_PI
Piper_nigrum_PI
PI_AT5G20240
Thottea_siliquosa_PI_3
Nymphaea_thermarum_PI_1
Nymphaea_thermarum_PI_2
Nymphaea_thermarum_PI_3
Amborella_trichopoda_PI
PI
Aristolochia_fimbriata_AP3
Aristolochia_ringens_AP3
Aristolochia_clematitis_AP3
Aristolochia_lindneri_AP3
Aristolochia_praevenosa_AP3
Aristolochia_deltantha_AP3
Aristolochia_arborea_AP3
Aristolochia_macrophylla_AP3_2
Aristolochia_manshuriensis_AP3_2
Aristolochia_macrophylla_AP3_1
Aristolochia_manshuriensis_AP3_1
Thottea_siliquosa_AP3
Asarum_canadense_AP3
Asarum_europaeum_AP3
Saruma_henryi_AP3
Peperomia_fraseri_PfAP3
Piper_nigrum_AP3_2c
Piper_methysticum_AP3
Piper_nigrum_AP3_2
Piper_nigrum_AP3_2b
Saururus_chinensis_AP3
Peperomia_hirta_PhAP3
Piper_nigrum_AP3_1
Houttuynia_cordata_AP3
AP3_AT3G54340
Hydnora_visseri_AP3
Amborella_trichopoda_AP3
Nymphaea_thermarum_AP3_1
Nymphaea_thermarum_AP3_2
Nymphaea_thermarum_AP3_3
AP3
Aristolochia_fimbriata_ABS_like
Aristolochia_clematitis_ABS_like
Aristolochia_deltantha_ABS_like1
Aristolochia_deltantha_ABS_like2
Aristolochia_praevenosa_ABS_like
Aristolochia_manshuriensis_ABS_like
Hydnora_visseri_ABS_like
Asarum_canadense_ABS_like
Asarum_europaeum_ABS_like
Saruma_henryi_ABS_like
Nymphaea_thermarum_ABS_like1
Nymphaea_thermarum_ABS_like2
Nymphaea_thermarum_ABS_like3
Piper_nigrum_ABS_like1
Piper_nigrum_ABS_like2
Amborella_trichopoda_ABS_like
ABS_AT5G23260
GOA_AT1G31140
ABS
0.3

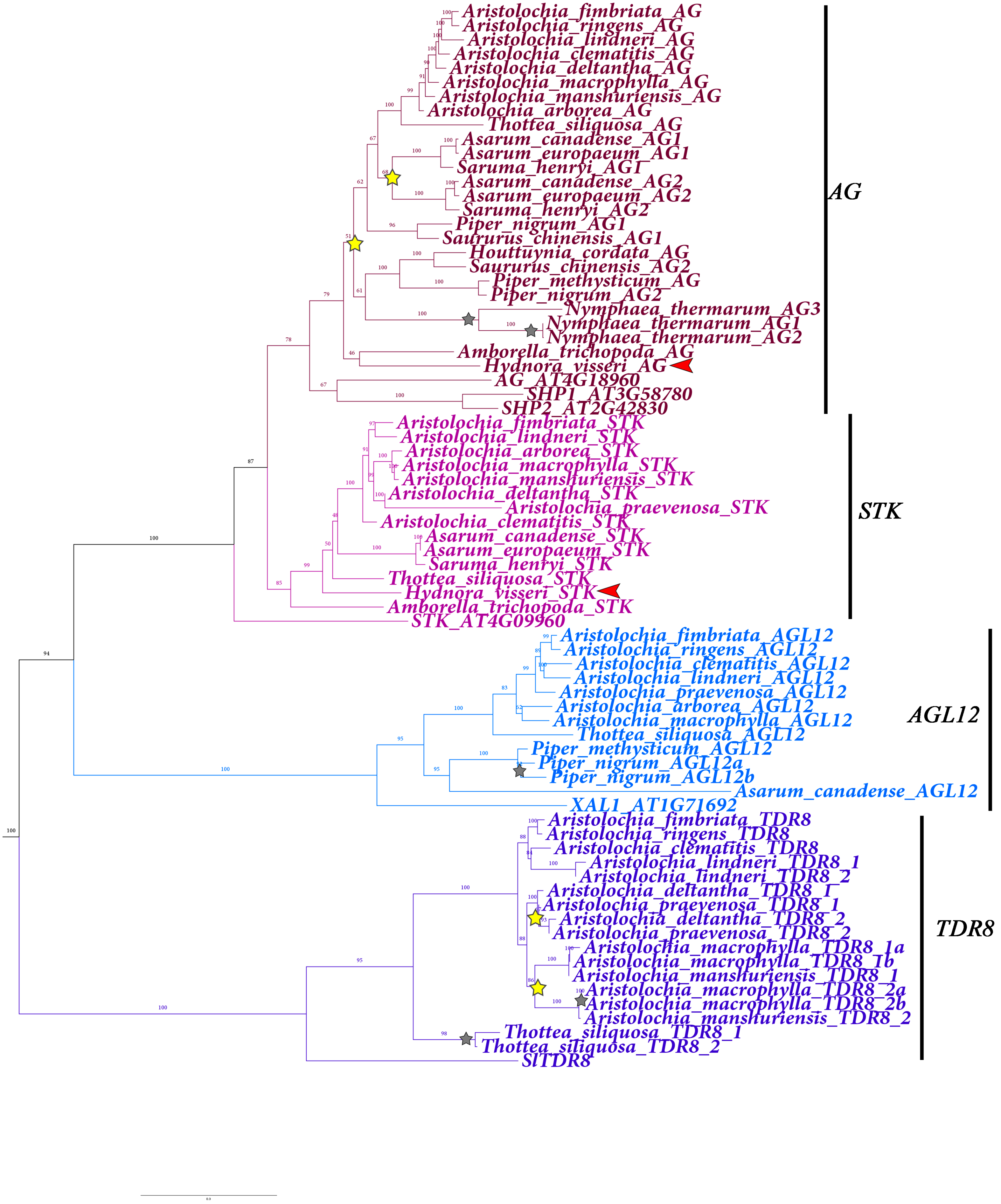

Aristolochia_fimbriata_AG
Aristolochia_ringens_AG
Aristolochia_lindneri_AG
Aristolochia_clematitis_AG
Aristolochia_deltantha_AG
Aristolochia_macrophylla_AG
Aristolochia_manshuriensis_AG
Aristolochia_arborea_AG
Thottea_siliquosa_AG
Asarum_canadense_AG1
Asarum_europaeum_AG1
Saruma_henryi_AG1
Asarum_canadense_AG2
Asarum_europaeum_AG2
Saruma_henryi_AG2
Piper_nigrum_AG1
Saururus_chinensis_AG1
Houttuynia_cordata_AG
Saururus_chinensis_AG2
Piper_methysticum_AG
Piper_nigrum_AG2
Nymphaea_thermarum_AG3
Nymphaea_thermarum_AG1
Nymphaea_thermarum_AG2
Amborella_trichopoda_AG
Hydnora_visseri_AG
AG_AT4G18960
SHP1_AT3G58780
SHP2_AT2G42830
AG
Aristolochia_fimbriata_STK
Aristolochia_lindneri_STK
Aristolochia_arborea_STK
Aristolochia_macrophylla_STK
Aristolochia_manshuriensis_STK
Aristolochia_deltantha_STK
Aristolochia_praevenosa_STK
Aristolochia_clematitis_STK
Asarum_canadense_STK
Asarum_europaeum_STK
Saruma_henryi_STK
Thottea_siliquosa_STK
Hydnora_visseri_STK
Amborella_trichopoda_STK
STK_AT4G09960
STK
Aristolochia_fimbriata_AGL12
Aristolochia_ringens_AGL12
Aristolochia_clematitis_AGL12
Aristolochia_lindneri_AGL12
Aristolochia_praevenosa_AGL12
Aristolochia_arborea_AGL12
Aristolochia_macrophylla_AGL12
Thottea_siliquosa_AGL12
Piper_methysticum_AGL12
Piper_nigrum_AGL12a
Piper_nigrum_AGL12b
Asarum_canadense_AGL12
XAL1_AT1G71692
AGL12
Aristolochia_fimbriata_TDR8
Aristolochia_ringens_TDR8
Aristolochia_clematitis_TDR8
Aristolochia_lindneri_TDR8_1
Aristolochia_lindneri_TDR8_2
Aristolochia_deltantha_TDR8_1
Aristolochia_praevenosa_TDR8_1
Aristolochia_deltantha_TDR8_2
Aristolochia_praevenosa_TDR8_2
Aristolochia_macrophylla_TDR8_1a
Aristolochia_macrophylla_TDR8_1b
Aristolochia_manshuriensis_TDR8_1
Aristolochia_macrophylla_TDR8_2a
Aristolochia_macrophylla_TDR8_2b
Aristolochia_manshuriensis_TDR8_2
Thottea_siliquosa_TDR8_1
Thottea_siliquosa_TDR8_2
SlTDR8
TDR8

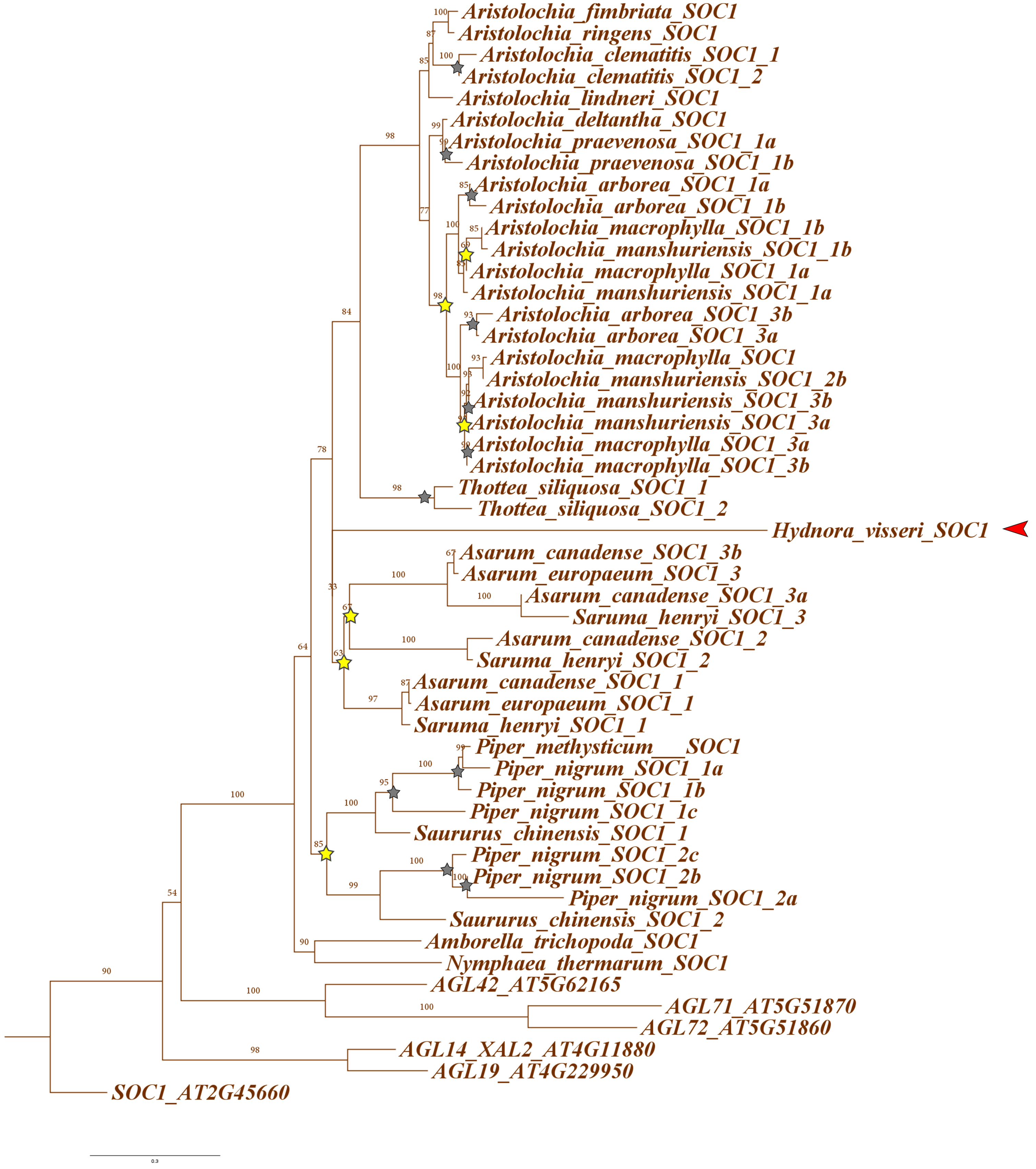

Aristolochia_fimbriata_SOC1
Aristolochia_ringens_SOC1
Aristolochia_clematitis_SOC1_1
Aristolochia_clematitis_SOC1_2
Aristolochia_lindneri_SOC1
Aristolochia_deltantha_SOC1
Aristolochia_praevenosa_SOC1_1a
Aristolochia_praevenosa_SOC1_1b
Aristolochia_arborea_SOC1_1a
Aristolochia_arborea_SOC1_1b
Aristolochia_macrophylla_SOC1_1b
Aristolochia_manshuriensis_SOC1_1b
Aristolochia_macrophylla_SOC1_1a
Aristolochia_manshuriensis_SOC1_1a
Aristolochia_arborea_SOC1_3b
Aristolochia_arborea_SOC1_3a
Aristolochia_macrophylla_SOC1
Aristolochia_manshuriensis_SOC1_2b
Aristolochia_manshuriensis_SOC1_3b
Aristolochia_manshuriensis_SOC1_3a
Aristolochia_macrophylla_SOC1_3a
Aristolochia_macrophylla_SOC1_3b
Thottea_siliquosa_SOC1_1
Thottea_siliquosa_SOC1_2
Hydnora_visseri_SOC1
Asarum_canadense_SOC1_3b
Asarum_europaeum_SOC1_3
Asarum_canadense_SOC1_3a
Saruma_henryi_SOC1_3
Asarum_canadense_SOC1_2
Saruma_henryi_SOC1_2
Asarum_canadense_SOC1_1
Asarum_europaeum_SOC1_1
Saruma_henryi_SOC1_1
Piper_methysticum___SOC1
Piper_nigrum_SOC1_1a
Piper_nigrum_SOC1_1b
Piper_nigrum_SOC1_1c
Saururus_chinensis_SOC1_1
Piper_nigrum_SOC1_2c
Piper_nigrum_SOC1_2b
Piper_nigrum_SOC1_2a
Saururus_chinensis_SOC1_2
Amborella_trichopoda_SOC1
Nymphaea_thermarum_SOC1
AGL42_AT5G62165
AGL71_AT5G51870
AGL72_AT5G51860
AGL14_XAL2_AT4G11880
AGL19_AT4G229950
SOC1_AT2G45660
0.3

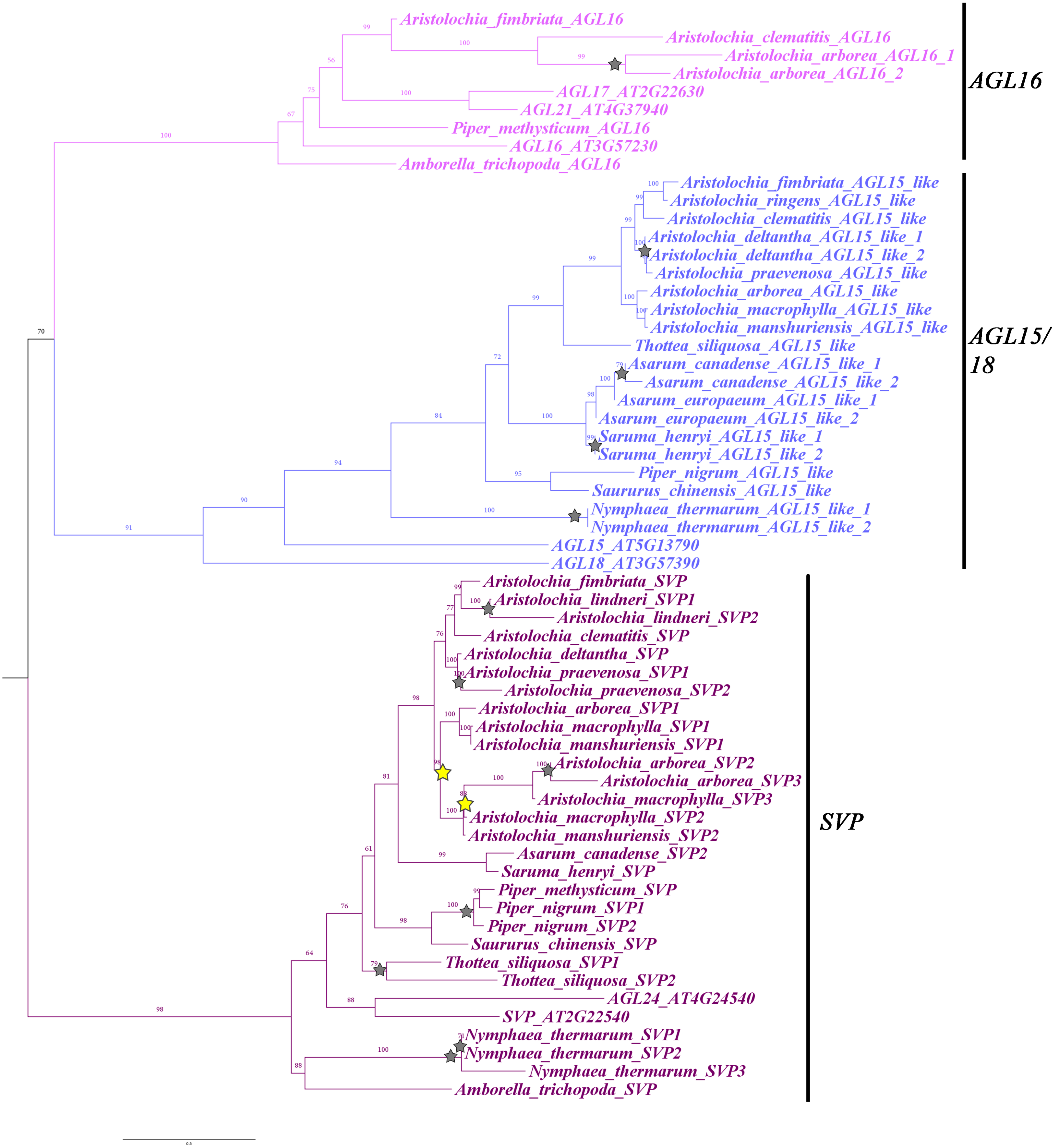
Aristolochia_fimbriata_AGL16
Aristolochia_clematitis_AGL16
Aristolochia_arborea_AGL16_1
Aristolochia_arborea_AGL16_2
AGL17_AT2G22630
AGL21_AT4G37940
Piper_methysticum_AGL16
AGL16_AT3G57230
Amborella_trichopoda_AGL16
AGL16
Aristolochia_fimbriata_AGL15_like
Aristolochia_ringens_AGL15_like
Aristolochia_clematitis_AGL15_like
Aristolochia_deltantha_AGL15_like_1
Aristolochia_deltantha_AGL15_like_2
Aristolochia_praevenosa_AGL15_like
Aristolochia_arborea_AGL15_like
Aristolochia_macrophylla_AGL15_like
Aristolochia_manshuriensis_AGL15_like
Thottea_siliquosa_AGL15_like
Asarum_canadense_AGL15_like_1
Asarum_canadense_AGL15_like_2
Asarum_europaeum_AGL15_like_1
Asarum_europaeum_AGL15_like_2
Saruma_henryi_AGL15_like_1
Saruma_henryi_AGL15_like_2
Piper_nigrum_AGL15_like
Saururus_chinensis_AGL15_like
Nymphaea_thermarum_AGL15_like_1
Nymphaea_thermarum_AGL15_like_2
AGL15_AT5G13790
AGL18_AT3G57390
AGL15/
18
Aristolochia_fimbriata_SVP
Aristolochia_lindneri_SVP1
Aristolochia_lindneri_SVP2
Aristolochia_clematitis_SVP
Aristolochia_deltantha_SVP
Aristolochia_praevenosa_SVP1
Aristolochia_praevenosa_SVP2
Aristolochia_arborea_SVP1
Aristolochia_macrophylla_SVP1
Aristolochia_manshuriensis_SVP1
Aristolochia_arborea_SVP2
Aristolochia_arborea_SVP3
Aristolochia_macrophylla_SVP3
Aristolochia_macrophylla_SVP2
Aristolochia_manshuriensis_SVP2
Asarum_canadense_SVP2
Saruma_henryi_SVP
Piper_methysticum_SVP
Piper_nigrum_SVP1
Piper_nigrum_SVP2
Saururus_chinensis_SVP
Thottea_siliquosa_SVP1
Thottea_siliquosa_SVP2
AGL24_AT4G24540
SVP_AT2G22540
Nymphaea_thermarum_SVP1
Nymphaea_thermarum_SVP2
Nymphaea_thermarum_SVP3
Amborella_trichopoda_SVP
SVP

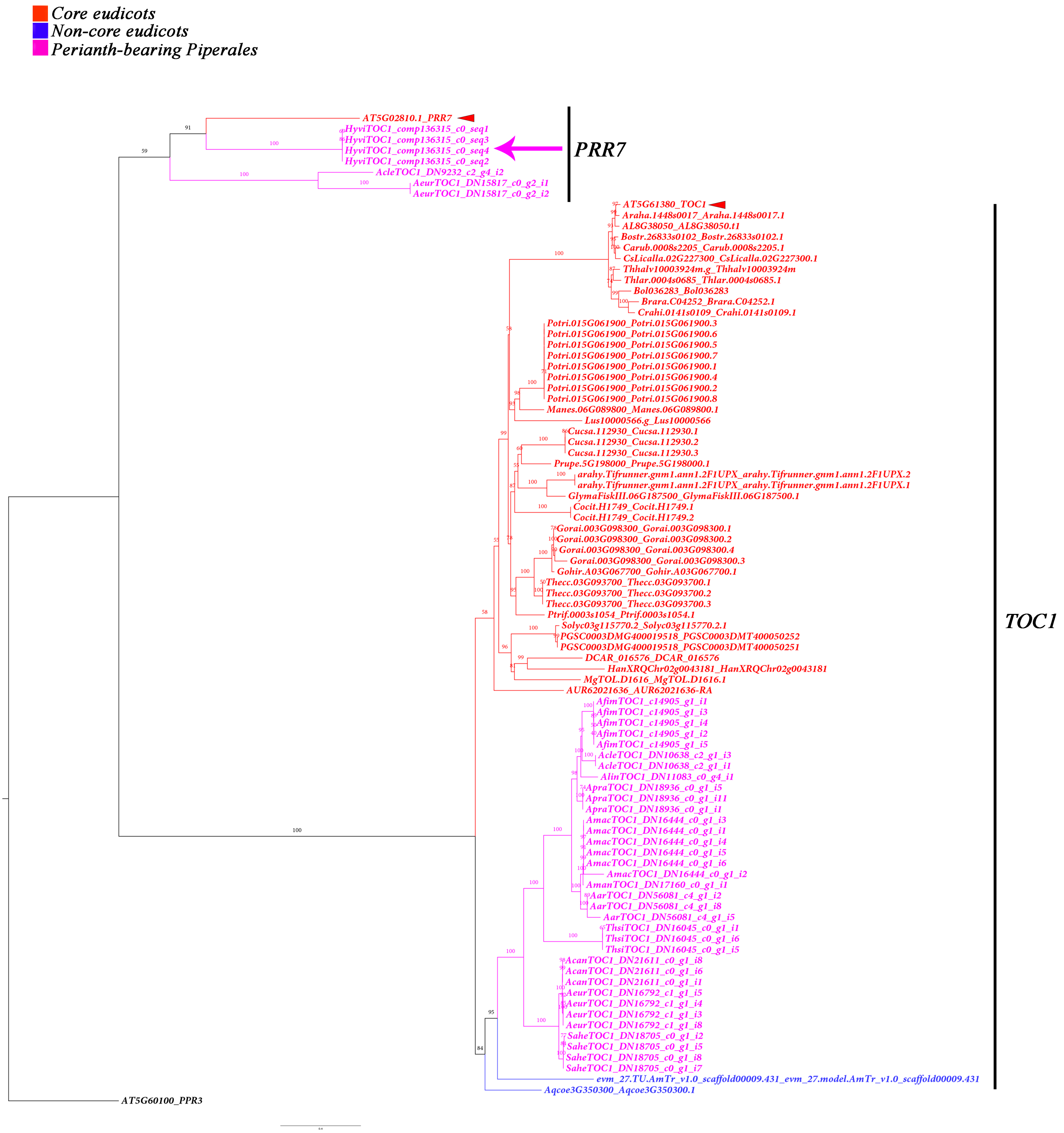

Core eudicots
Non-core eudicots
Perianth-bearing Piperales
PRR7
TOC1
AT5G02810.1_PRR7
HyviTOC1_comp136315_c0_seq1
HyviTOC1_comp136315_c0_seq3
HyviTOC1_comp136315_c0_seq4
HyviTOC1_comp136315_c0_seq2
AcleTOC1_DN9232_c2_g4_i2
AeurTOC1_DN15817_c0_g2_i1
AeurTOC1_DN15817_c0_g2_i2
AT5G61380_TOC1
Araha.1448s0017_Araha.1448s0017.1
AL8G38050_AL8G38050.t1
Bostr.26833s0102_Bostr.26833s0102.1
Carub.0008s2205_Carub.0008s2205.1
CsLicalla.02G227300_CsLicalla.02G227300.1
Thhalv10003924m.g_Thhalv10003924m
Thlar.0004s0685_Thlar.0004s0685.1
Bol036283_Bol036283
Brara.C04252_Brara.C04252.1
Crahi.0141s0109_Crahi.0141s0109.1
Potri.015G061900_Potri.015G061900.3
Potri.015G061900_Potri.015G061900.6
Potri.015G061900_Potri.015G061900.5
Potri.015G061900_Potri.015G061900.7
Potri.015G061900_Potri.015G061900.1
Potri.015G061900_Potri.015G061900.4
Potri.015G061900_Potri.015G061900.2
Potri.015G061900_Potri.015G061900.8
Manes.06G089800_Manes.06G089800.1
Lus10000566.g_Lus10000566
Cucsa.112930_Cucsa.112930.1
Cucsa.112930_Cucsa.112930.2
Cucsa.112930_Cucsa.112930.3
Prupe.5G198000_Prupe.5G198000.1
arahy.Tifrunner.gnm1.ann1.2F1UPX_arahy.Tifrunner.gnm1.ann1.2F1UPX.2
arahy.Tifrunner.gnm1.ann1.2F1UPX_arahy.Tifrunner.gnm1.ann1.2F1UPX.1
GlymaFiskIII.06G187500_GlymaFiskIII.06G187500.1
Cocit.H1749_Cocit.H1749.1
Cocit.H1749_Cocit.H1749.2
Gorai.003G098300_Gorai.003G098300.1
Gorai.003G098300_Gorai.003G098300.2
Gorai.003G098300_Gorai.003G098300.4
Gorai.003G098300_Gorai.003G098300.3
Gohir.A03G067700_Gohir.A03G067700.1
Thecc.03G093700_Thecc.03G093700.1
Thecc.03G093700_Thecc.03G093700.2
Thecc.03G093700_Thecc.03G093700.3
Ptrif.0003s1054_Ptrif.0003s1054.1
Solyc03g115770.2_Solyc03g115770.2.1
PGSC0003DMG400019518_PGSC0003DMT400050252
PGSC0003DMG400019518_PGSC0003DMT400050251
DCAR_016576_DCAR_016576
HanXRQChr02g0043181_HanXRQChr02g0043181
MgTOL.D1616_MgTOL.D1616.1
AUR62021636_AUR62021636-RA
AfimTOC1_c14905_g1_i1
AfimTOC1_c14905_g1_i3
AfimTOC1_c14905_g1_i4
AfimTOC1_c14905_g1_i2
AfimTOC1_c14905_g1_i5
AcleTOC1_DN10638_c2_g1_i3
AcleTOC1_DN10638_c2_g1_i1
AlinTOC1_DN11083_c0_g4_i1
ApraTOC1_DN18936_c0_g1_i5
ApraTOC1_DN18936_c0_g1_i11
ApraTOC1_DN18936_c0_g1_i1
AmacTOC1_DN16444_c0_g1_i3
AmacTOC1_DN16444_c0_g1_i1
AmacTOC1_DN16444_c0_g1_i4
AmacTOC1_DN16444_c0_g1_i5
AmacTOC1_DN16444_c0_g1_i6
AmacTOC1_DN16444_c0_g1_i2
AmanTOC1_DN17160_c0_g1_i1
AarTOC1_DN56081_c4_g1_i2
AarTOC1_DN56081_c4_g1_i8
AarTOC1_DN56081_c4_g1_i5
ThsiTOC1_DN16045_c0_g1_i1
ThsiTOC1_DN16045_c0_g1_i6
ThsiTOC1_DN16045_c0_g1_i5
AcanTOC1_DN21611_c0_g1_i8
AcanTOC1_DN21611_c0_g1_i6
AcanTOC1_DN21611_c0_g1_i1
AeurTOC1_DN16792_c1_g1_i5
AeurTOC1_DN16792_c1_g1_i4
AeurTOC1_DN16792_c1_g1_i3
AeurTOC1_DN16792_c1_g1_i8
SaheTOC1_DN18705_c0_g1_i2
SaheTOC1_DN18705_c0_g1_i5
SaheTOC1_DN18705_c0_g1_i8
SaheTOC1_DN18705_c0_g1_i7
evm_27.TU.AmTr_v1.0_scaffold00009.431_evm_27.model.AmTr_v1.0_scaffold00009.431
Aqcoe3G350300_Aqcoe3G350300.1
AT5G60100_PPR3

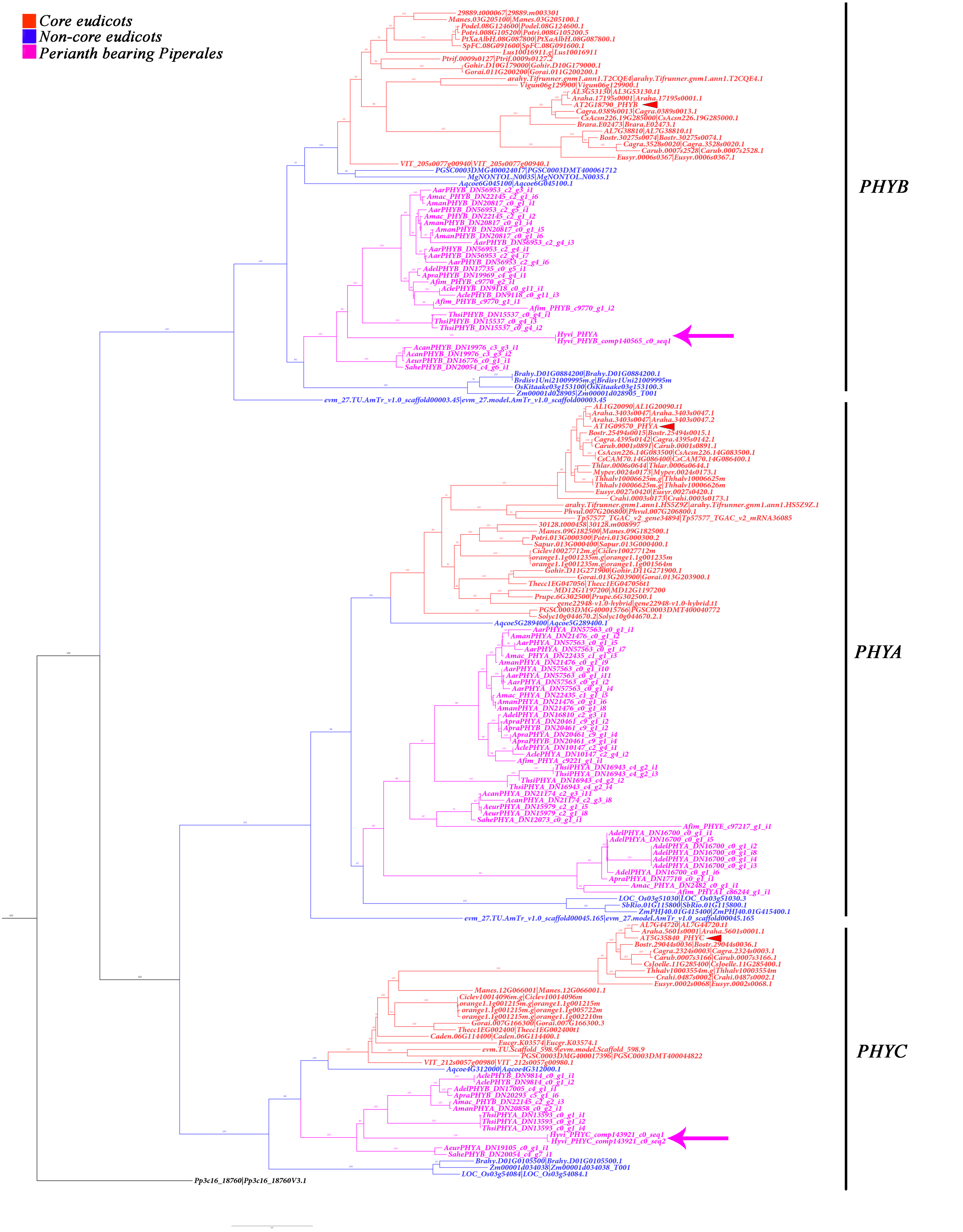

Core eudicots
Non-core eudicots
Perianth bearing Piperales
PHYB
PHYA
PHYC
29889.t000067|29889.m003301
Manes.03G205100|Manes.03G205100.1
Podel.08G124600|Podel.08G124600.1
Potri.008G105200|Potri.008G105200.5
PtXaAlbH.08G087800|PtXaAlbH.08G087800.1
SpFC.08G091600|SpFC.08G091600.1
Lus10016911.g|Lus10016911
Ptrif.0009s0127|Ptrif.0009s0127.2
Gohir.D10G179000|Gohir.D10G179000.1
Gorai.011G200200|Gorai.011G200200.1
arahy.Tifrunner.gnm1.ann1.T2CQE4|arahy.Tifrunner.gnm1.ann1.T2CQE4.1
Vigun06g129900|Vigun06g129900.1
AL3G53130|AL3G53130.t1
Araha.17195s0001|Araha.17195s0001.1
AT2G18790_PHYB
Cagra.0389s0013|Cagra.0389s0013.1
CsAcsn226.19G285000|CsAcsn226.19G285000.1
Brara.E02473|Brara.E02473.1
AL7G38810|AL7G38810.t1
Bostr.30275s0074|Bostr.30275s0074.1
Cagra.3528s0020|Cagra.3528s0020.1
Carub.0007s2528|Carub.0007s2528.1
Eusyr.0006s0367|Eusyr.0006s0367.1
VIT_205s0077g00940|VIT_205s0077g00940.1
PGSC0003DMG400024017|PGSC0003DMT400061712
MgNONTOL.N0035|MgNONTOL.N0035.1
Aqcoe6G045100|Aqcoe6G045100.1
AarPHYB_DN56953_c2_g3_i1
Amac_PHYB_DN22145_c2_g1_i6
AmanPHYB_DN20817_c0_g1_i1
AarPHYB_DN56953_c2_g5_i1
Amac_PHYB_DN22145_c2_g1_i2
AmanPHYB_DN20817_c0_g1_i4
AmanPHYB_DN20817_c0_g1_i5
AmanPHYB_DN20817_c0_g1_i6
AarPHYB_DN56953_c2_g4_i3
AarPHYB_DN56953_c2_g4_i1
AarPHYB_DN56953_c2_g4_i7
AarPHYB_DN56953_c2_g4_i6
AdelPHYB_DN17735_c0_g5_i1
ApraPHYB_DN19969_c4_g4_i1
Afim_PHYB_c9770_g2_i1
AclePHYB_DN9118_c0_g11_i1
AclePHYB_DN9118_c0_g11_i3
Afim_PHYB_c9770_g1_i1
Afim_PHYB_c9770_g1_i2
ThsiPHYB_DN15537_c0_g4_i1
ThsiPHYB_DN15537_c0_g4_i3
ThsiPHYB_DN15537_c0_g4_i2
Hyvi_PHYA
Hyvi_PHYB_comp140565_c0_seq1
AcanPHYB_DN19976_c3_g3_i1
AcanPHYB_DN19976_c3_g3_i2
AeurPHYB_DN16776_c0_g1_i1
SahePHYB_DN20054_c4_g6_i1
Brahy.D01G0884200|Brahy.D01G0884200.1
Brdisv1Uni21009995m.g|Brdisv1Uni21009995m
OsKitaake03g153100|OsKitaake03g153100.3
Zm00001d028905|Zm00001d028905_T001
evm_27.TU.AmTr_v1.0_scaffold00003.45|evm_27.model.AmTr_v1.0_scaffold00003.45
AL1G20090|AL1G20090.t1
Araha.3403s0047|Araha.3403s0047.1
Araha.3403s0047|Araha.3403s0047.2
AT1G09570_PHYA
Bostr.25494s0015|Bostr.25494s0015.1
Cagra.4395s0142|Cagra.4395s0142.1
Carub.0001s0891|Carub.0001s0891.1
CsAcsn226.14G083500|CsAcsn226.14G083500.1
CsCAM70.14G086400|CsCAM70.14G086400.1
Thlar.0006s0644|Thlar.0006s0644.1
Myper.0024s0173|Myper.0024s0173.1
Thhalv10006625m.g|Thhalv10006625m
Thhalv10006625m.g|Thhalv10006626m
Eusyr.0027s0420|Eusyr.0027s0420.1
Crahi.0003s0173|Crahi.0003s0173.1
arahy.Tifrunner.gnm1.ann1.HS5Z9Z|arahy.Tifrunner.gnm1.ann1.HS5Z9Z.1
Phvul.007G206800|Phvul.007G206800.1
Tp57577_TGAC_v2_gene34894|Tp57577_TGAC_v2_mRNA36085
30128.t000458|30128.m008997
Manes.09G182500|Manes.09G182500.1
Potri.013G000300|Potri.013G000300.2
Sapur.013G000400|Sapur.013G000400.1
Ciclev10027712m.g|Ciclev10027712m
orange1.1g001235m.g|orange1.1g001235m
orange1.1g001235m.g|orange1.1g001564m
Gohir.D11G271900|Gohir.D11G271900.1
Gorai.013G203900|Gorai.013G203900.1
Thecc1EG047056|Thecc1EG047056t1
MD12G1197200|MD12G1197200
Prupe.6G302500|Prupe.6G302500.1
gene22948-v1.0-hybrid|gene22948-v1.0-hybrid.t1
PGSC0003DMG400015766|PGSC0003DMT400040772
Solyc10g044670.2|Solyc10g044670.2.1
Aqcoe5G289400|Aqcoe5G289400.1
AarPHYA_DN57563_c0_g1_i1
AmanPHYA_DN21476_c0_g1_i2
AarPHYA_DN57563_c0_g1_i5
AarPHYA_DN57563_c0_g1_i7
Amac_PHYA_DN22435_c1_g1_i3
AmanPHYA_DN21476_c0_g1_i9
AarPHYA_DN57563_c0_g1_i10
AarPHYA_DN57563_c0_g1_i11
AarPHYA_DN57563_c0_g1_i2
AarPHYA_DN57563_c0_g1_i4
Amac_PHYA_DN22435_c1_g1_i5
AmanPHYA_DN21476_c0_g1_i6
AmanPHYA_DN21476_c0_g1_i8
AdelPHYA_DN16810_c2_g3_i1
ApraPHYA_DN20461_c9_g1_i2
ApraPHYB_DN20461_c9_g1_i2
ApraPHYA_DN20461_c9_g1_i4
ApraPHYB_DN20461_c9_g1_i4
AclePHYA_DN10147_c2_g4_i1
AclePHYA_DN10147_c2_g4_i2
Afim_PHYA_c9221_g1_i1
ThsiPHYA_DN16943_c4_g2_i1
ThsiPHYA_DN16943_c4_g2_i3
ThsiPHYA_DN16943_c4_g2_i2
ThsiPHYA_DN16943_c4_g2_i4
AcanPHYA_DN21174_c2_g3_i11
AcanPHYA_DN21174_c2_g3_i8
AeurPHYA_DN15979_c2_g1_i5
AeurPHYA_DN15979_c2_g1_i8
SahePHYA_DN12073_c0_g1_i1
Afim_PHYE_c97217_g1_i1
AdelPHYA_DN16700_c0_g1_i1
AdelPHYA_DN16700_c0_g1_i5
AdelPHYA_DN16700_c0_g1_i2
AdelPHYA_DN16700_c0_g1_i8
AdelPHYA_DN16700_c0_g1_i4
AdelPHYA_DN16700_c0_g1_i3
AdelPHYA_DN16700_c0_g1_i6
ApraPHYA_DN17710_c0_g1_i1
Amac_PHYA_DN2482_c0_g1_i1
Afim_PHYA1_c86244_g1_i1
LOC_Os03g51030|LOC_Os03g51030.3
SbRio.01G115800|SbRio.01G115800.1
ZmPHJ40.01G415400|ZmPHJ40.01G415400.1
evm_27.TU.AmTr_v1.0_scaffold00045.165|evm_27.model.AmTr_v1.0_scaffold00045.165
AL7G44720|AL7G44720.t1
Araha.5601s0001|Araha.5601s0001.1
AT5G35840_PHYC
Bostr.29044s0036|Bostr.29044s0036.1
Cagra.2324s0003|Cagra.2324s0003.1
Carub.0007s3166|Carub.0007s3166.1
CsJoelle.11G285400|CsJoelle.11G285400.1
Thhalv10003554m.g|Thhalv10003554m
Crahi.0487s0002|Crahi.0487s0002.1
Eusyr.0002s0068|Eusyr.0002s0068.1
Manes.12G066001|Manes.12G066001.1
Ciclev10014096m.g|Ciclev10014096m
orange1.1g001215m.g|orange1.1g001215m
orange1.1g001215m.g|orange1.1g005722m
orange1.1g001215m.g|orange1.1g002210m
Gorai.007G166300|Gorai.007G166300.3
Thecc1EG002400|Thecc1EG002400t1
Caden.06G114400|Caden.06G114400.1
Eucgr.K03574|Eucgr.K03574.1
evm.TU.Scaffold_598.9|evm.model.Scaffold_598.9
PGSC0003DMG400017396|PGSC0003DMT400044822
VIT_212s0057g00980|VIT_212s0057g00980.1
Aqcoe4G312000|Aqcoe4G312000.1
AclePHYB_DN9814_c0_g1_i1
AclePHYB_DN9814_c0_g1_i2
AdelPHYB_DN17005_c4_g1_i1
ApraPHYB_DN20293_c5_g1_i6
Amac_PHYB_DN22145_c2_g2_i3
AmanPHYA_DN20858_c0_g2_i1
ThsiPHYA_DN13593_c0_g1_i1
ThsiPHYA_DN13593_c0_g1_i2
ThsiPHYA_DN13593_c0_g1_i4
Hyvi_PHYC_comp143921_c0_seq1
Hyvi_PHYC_comp143921_c0_seq2
AeurPHYA_DN19105_c0_g1_i1
SahePHYB_DN20054_c4_g7_i1
Brahy.D01G0105500|Brahy.D01G0105500.1
Zm00001d034038|Zm00001d034038_T001
LOC_Os03g54084|LOC_Os03g54084.1
Pp3c16_18760|Pp3c16_18760V3.1

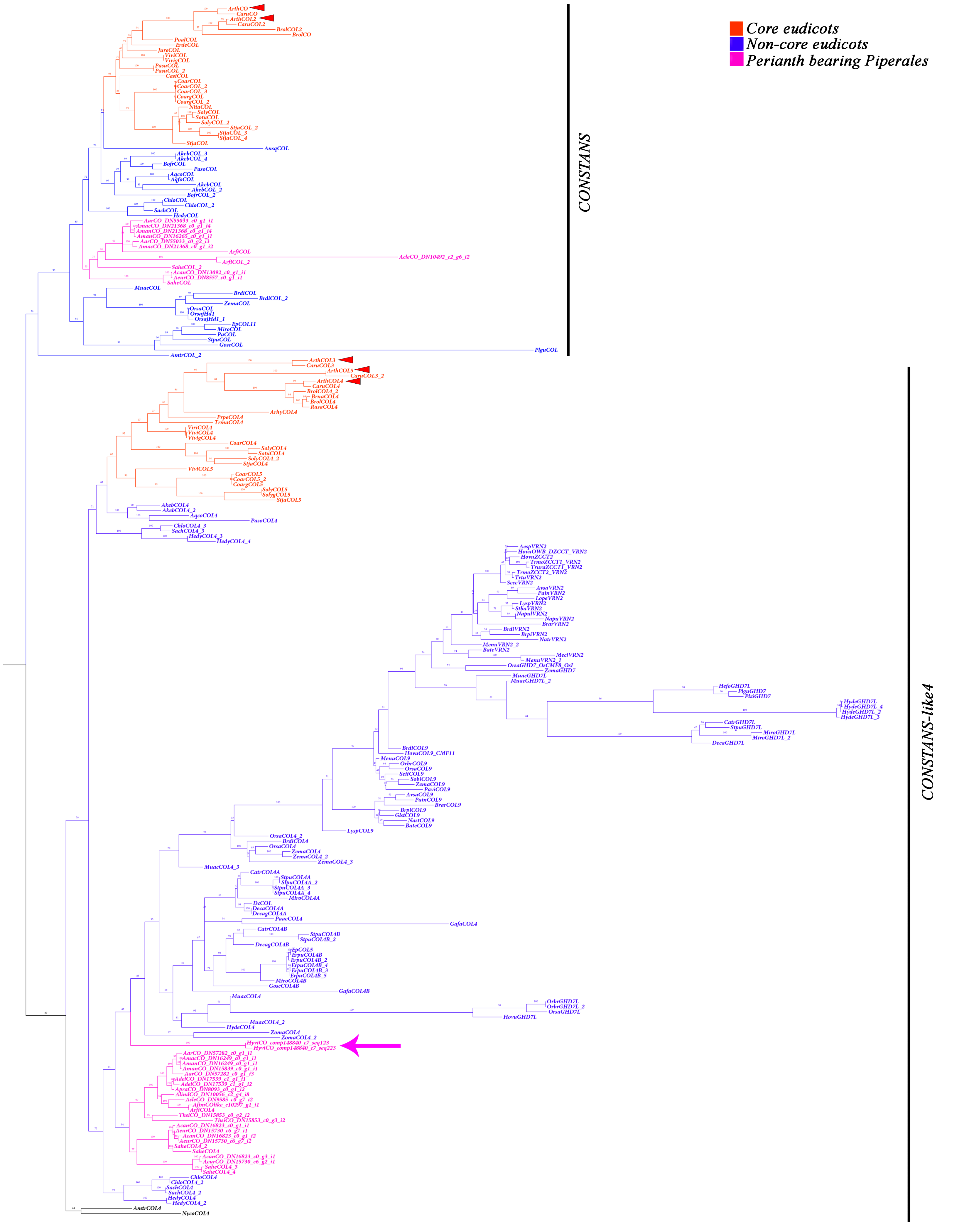

Core eudicots
Non-core eudicots
Perianth bearing Piperales
CONSTANS
CONSTANS-like4
ArthCO
CaruCO
ArthCOL2
CaruCOL2
BrolCOL2
BrolCO
PoalCOL
ErdeCOL
JureCOL
ViviCOL
VivigCOL
PasuCOL
PasuCOL_2
CasiCOL
CoarCOL
CoarCOL_2
CoarCOL_3
CoargCOL
CoargCOL_2
NitaCOL
SolyCOL
SotuCOL
SolyCOL_2
StjaCOL_2
StjaCOL_3
StjaCOL_4
StjaCOL
AnsqCOL
AkebCOL_3
AkebCOL_4
BofrCOL
PasoCOL
AqcoCOL
AqfoCOL
AkebCOL
AkebCOL_2
BofrCOL_2
ChloCOL
ChloCOL_2
SachCOL
HedyCOL
AarCO_DN55033_c0_g1_i1
AmacCO_DN21368_c0_g1_i4
AmanCO_DN21368_c0_g1_i4
AmanCO_DN16265_c0_g1_i1
AarCO_DN55033_c0_g2_i3
AmacCO_DN21368_c0_g1_i2
ArfiCOL
AcleCO_DN10492_c2_g6_i2
ArfiCOL_2
SaheCOL_2
AcanCO_DN13092_c0_g1_i1
AeurCO_DN8557_c0_g1_i1
SaheCOL
MuacCOL
BrdiCOL
BrdiCOL_2
ZemaCOL
OrsaCOL
OrsajHd1
OrsajHd1_1
EpCOL11
MiroCOL
PaCOL
StpuCOL
GoscCOL
PlguCOL
AmtrCOL_2
ArthCOL3
CaruCOL3
ArthCOL5
CaruCOL3_2
ArthCOL4
CaruCOL4
BrolCOL4_2
BrnaCOL4
BrolCOL4
RasaCOL4
ArhyCOL4
PrpeCOL4
TrmaCOL4
ViriCOL4
ViviCOL4
VivigCOL4
CoarCOL4
SolyCOL4
SotuCOL4
SolyCOL4_2
StjaCOL4
ViviCOL5
CoarCOL5
CoarCOL5_2
CoargCOL5
SolyCOL5
SolygCOL5
StjaCOL5
AkebCOL4
AkebCOL4_2
AqcoCOL4
PasoCOL4
ChloCOL4_3
SachCOL4_3
HedyCOL4_3
HedyCOL4_4
AespVRN2
HovuOWB_DZCCT_VRN2
HovuZCCT2
TrmoZCCT1_VRN2
TruraZCCT1_VRN2
TrmoZCCT2_VRN2
TrtuVRN2
SeceVRN2
AvsaVRN2
PainVRN2
LopeVRN2
LyspVRN2
StbaVRN2
NapulVRN2
NapuVRN2
BrarVRN2
BrdiVRN2
BrpiVRN2
NatrVRN2
MenuVRN2_2
BateVRN2
MeciVRN2
MenuVRN2_1
OrsaGHD7_OsCMF8_OsI
ZemaGHD7
MuacGHD7L
MuacGHD7L_2
HefoGHD7L
PlguGHD7
PlziGHD7
HydeGHD7L
HydeGHD7L_4
HydeGHD7L_2
HydeGHD7L_3
CatrGHD7L
StpuGHD7L
MiroGHD7L
MiroGHD7L_2
DecaGHD7L
BrdiCOL9
HovuCOL9_CMF11
MenuCOL9
OrbrCOL9
OrsaCOL9
SeitCOL9
SobiCOL9
ZemaCOL9
PaviCOL9
AvsaCOL9
PainCOL9
BrarCOL9
BrpiCOL9
GlstCOL9
NastCOL9
BateCOL9
LyspCOL9
OrsaCOL4_2
BrdiCOL4
OrsaCOL4
ZemaCOL4
ZemaCOL4_2
ZemaCOL4_3
MuacCOL4_3
CatrCOL4A
StpuCOL4A
StpuCOL4A_2
StpuCOL4A_3
StpuCOL4A_4
MiroCOL4A
DcCOL
DecaCOL4A
DecagCOL4A
PaaeCOL4
GafaCOL4
CatrCOL4B
StpuCOL4B
StpuCOL4B_2
DecagCOL4B
EpCOL5
ErpuCOL4B
ErpuCOL4B_2
ErpuCOL4B_4
ErpuCOL4B_3
ErpuCOL4B_5
MiroCOL4B
GoscCOL4B
GafaCOL4B
MuacCOL4
OrbrGHD7L
OrbrGHD7L_2
OrsaGHD7L
HovuGHD7L
MuacCOL4_2
HydeCOL4
ZomaCOL4
ZomaCOL4_2
HyviCO_comp148840_c7_seq123
HyviCO_comp148840_c7_seq223
AarCO_DN57282_c0_g1_i1
AmacCO_DN16249_c0_g1_i1
AmanCO_DN16249_c0_g1_i1
AmanCO_DN15839_c0_g1_i1
AarCO_DN57282_c0_g1_i3
AdelCO_DN17539_c1_g1_i1
AdelCO_DN17539_c1_g1_i2
ApraCO_DN8093_c0_g1_i2
AlindCO_DN10056_c2_g4_i8
AcleCO_DN9585_c0_g7_i2
AfimCOlike_c10297_g1_i1
ArfiCOL4
ThsiCO_DN15853_c0_g2_i2
ThsiCO_DN15853_c0_g3_i2
AcanCO_DN16823_c0_g1_i1
AeurCO_DN15730_c6_g7_i1
AcanCO_DN16823_c0_g1_i2
AeurCO_DN15730_c6_g7_i2
SaheCOL4_2
SaheCOL4
AcanCO_DN16823_c0_g3_i1
AeurCO_DN15730_c6_g2_i1
SaheCOL4_3
SaheCOL4_4
ChloCOL4
ChloCOL4_2
SachCOL4
SachCOL4_2
HedyCOL4
HedyCOL4_2
AmtrCOL4
NycoCOL4
